# The Analysis of Field Strains Isolated From Food, Animal and Clinical Sources Uncovers Natural Mutations in *Listeria monocytogenes* Nisin Resistance Genes

**DOI:** 10.3389/fmicb.2020.549531

**Published:** 2020-10-06

**Authors:** Joseph Wambui, Athmanya K. Eshwar, Mariella Aalto-Araneda, Anna Pöntinen, Marc J. A. Stevens, Patrick M. K. Njage, Taurai Tasara

**Affiliations:** ^1^Institute for Food Safety and Hygiene, Vetsuisse Faculty, University of Zurich, Zurich, Switzerland; ^2^Graduate School for Cellular and Biomedical Sciences, University of Bern, Bern, Switzerland; ^3^Department of Food Hygiene and Environmental Health, Faculty of Veterinary Medicine, University of Helsinki, Helsinki, Finland; ^4^Research Group for Genomic Epidemiology, Division for Global Surveillance, National Food Institute, Technical University of Denmark, Kengens Lyngby, Denmark

**Keywords:** *Listeria monocytogenes*, bacteriocin, nisin, resistance, mutation, genes

## Abstract

Nisin is a commonly used bacteriocin for controlling spoilage and pathogenic bacteria in food products. Strains possessing high natural nisin resistance that reduce or increase the potency of this bacteriocin against *Listeria monocytogenes* have been described. Our study sought to gather more insights into nisin resistance mechanisms in natural *L. monocytogenes* populations by examining a collection of 356 field strains that were isolated from different foods, food production environments, animals and human infections. A growth curve analysis-based approach was used to access nisin inhibition levels and assign the *L. monocytogenes* strains into three nisin response phenotypic categories; resistant (66%), intermediate (26%), and sensitive (8%). Using this categorization isolation source, serotype, genetic lineage, clonal complex (CC) and strain-dependent natural variation in nisin phenotypic resistance among *L. monocytogenes* field strains was revealed. Whole genome sequence analysis and comparison of high nisin resistant and sensitive strains led to the identification of new naturally occurring mutations in nisin response genes associated with increased nisin resistance and sensitivity in this bacterium. Increased nisin resistance was detected in strains harboring RsbU_G77S_ and PBPB3_V240F_ amino acid substitution mutations, which also showed increased detergent stress resistance as well as increased virulence in a zebra fish infection model. On the other hand, increased natural nisin sensitivity was detected among strains with mutations in *sigB*, *vir*, and *dlt* operons that also showed increased lysozyme sensitivity and lower virulence. Overall, our study identified naturally selected mutations involving *pbpB3* (*lm0441*) as well as *sigB*, *vir*, and *dlt* operon genes that are associated with intrinsic nisin resistance in *L. monocytogenes* field strains recovered from various food and human associated sources. Finally, we show that combining growth parameter-based phenotypic analysis and genome sequencing is an effective approach that can be useful for the identification of novel nisin response associated genetic variants among *L. monocytogenes* field strains.

## Introduction

*Listeria monocytogenes*, the cause of listeriosis, constitutes an important foodborne pathogen with significant public health and food-safety problems. Listeriosis has serious clinical consequences due to its high mortality rate in people with weakened immune systems, including children, pregnant women and the elderly individuals (Kayode et al., 2019; Maia et al., 2019). The majority of foodborne outbreaks and large-scale food recalls that are instituted continue to be caused by food product contamination with *L. monocytogenes* (Cabal et al., 2019; Ly et al., 2019; Nichols et al., 2019). Risks emanating from *L. monocytogenes* contamination in food are attributed to the widespread occurrence and capability of this bacterium to survive, persist and proliferate on various types of food and in food-associated processing and production environments (Bucur et al., 2018; Rodríguez-López et al., 2018). Such attributes emanate from various molecular and physiological response mechanisms enabling adaptation and resistance of this bacterium against stress conditions intrinsic to different food types or imposed through food preservation measures (Harter et al., 2017).

One of the widely used strategies against *L. monocytogenes* in food involves inclusion of nisin, a positively charged antimicrobial peptide produced by some strains of *Lactococcus lactis* subsp. *lactis* (Cotter et al., 2005; Zhou et al., 2014; Alvarez-Sieiro et al., 2016). Due to its stability, broad specificity and low toxicity, nisin has been classified as GRAS (Generally Recognized as Safe) and gained wide application as a food preservative for various food types (Bali et al., 2016; Santos et al., 2018). Different food safety authorities have different standards for the accepted concentration of nisin in foods but its general application around the globe, and specifically in milk and milk products, is regulated by the FAO/WHO Codex Committee that has set 12.5 ppm as the acceptable concentration in the products (Ross et al., 2002; Müller-Auffermann et al., 2015). Nisin has two modes of antimicrobial activity that are generally recognized in the Gram-positive bacteria. First, it inhibits peptidoglycan biosynthesis by binding to the precursor lipid II. This leads to formation of nisin-lipid II aggregates that are encapsulated on the membranes, a process which results in deformation of the cell membrane and ultimately cell death (Breukink et al., 1999; Wiedemann et al., 2001; Scherer et al., 2015). Secondly, nisin targets the cell membrane where it forms pores resulting in leakage and ultimately cell autolysis due to inactivation of various cellular reactions (McAuliffe et al., 2001). The disruption of the cell envelope structure occurs due to nisin’s positive charge that facilitates its adsorption to the negatively charged cell envelope surface (Martin et al., 1996).

The effective use of nisin against *L. monocytogenes* is hampered by innate and acquired resistance mechanisms. Nisin resistance in this bacterium has been attributed to alteration of the cell wall composition and charges thus preventing nisin from accessing lipid II (Bucur et al., 2018). The response mechanisms involved include the D-alanylation of teichoic acids in the cell wall by enzymes encoded in the *dlt* operon, which increases net positive charge and cell wall density (Abachin et al., 2002; Saar-Dover et al., 2012). Mutants lacking components of the *dlt* operon have a more negatively charged, thin cell wall that binds more effectively to the positively charged nisin (Peschel et al., 1999; Baddiley, 2000). The cell envelope charges can also be altered through the MprF mediated lysylation of the cell membrane phospholipids (Thedieck et al., 2006; Kang et al., 2015). Alteration of the net cell surface charge is regulated by VirRS, a two components system (TCS) that has not only been shown to be important for innate response to nisin in *L. monocytogenes* but it regulates key nisin resistance genes among them *dltABCD*, *mprF*, and *anrAB* (Mandin et al., 2005; Collins et al., 2010a; Kang et al., 2015). AnrAB are ABC transporters that are thought to promote intrinsic resistance through export of nisin across the cell membrane (Collins et al., 2010a). The regulation of nisin response genes by VirRS requires the VirAB ABC transporters, whose primary function is sensing and signaling the presence of nisin (Grubaugh et al., 2018). On the other hand, the TCS CesRK and LisRK are also involved in sensing and responding to nisin by regulating cell envelope-related genes among the penicillin binding proteins (PBPs) Lmo0441 (PBPB3) and Lmo2229 (PBPA2), respectively (Cotter et al., 2002; Kallipolitis et al., 2003; Gottschalk et al., 2008). LisRK is also a positive regulator of *lmo1021* gene that encodes LiaS, which is part of the three-component system LiaFSR that also mediates nisin response (Cotter et al., 2002; Fritsch et al., 2011). The LisK-dependent expression of *lmo0441* occurs in a LiaS-dependent manner whereas LiaR regulates *virB* expression (Gravesen et al., 2001, 2004; Bergholz et al., 2013). Therefore, there seems to be a regulatory cascade loop for the various systems that are involved in nisin response in *L. monocytogenes*. Other genes known to contribute to nisin through yet undefined mechanisms include *gad*D1 (Begley et al., 2010) and *tel*A (Collins et al., 2010b). Regulatory proteins RsbU and the alternative sigma factors, SigB, SigL, and SigC also contribute to nisin response through mechanisms that are yet to be fully understood (Zhang et al., 2005; Begley et al., 2006; Palmer et al., 2009; Shin et al., 2010).

Previously, various approaches have been used to identify specific nisin resistance mechanisms and interrelationship between various genes in *L. monocytogenes* including screening of transposon library mutants (Collins et al., 2010a,b) and transcriptome analysis (Wu et al., 2018), but based on a few laboratory based strains. More recently, a combination of minimum inhibitory concentration and whole genome sequencing approach on six strains derived from the screening of 282 *L. monocytogenes* field isolates was used after which acquired nisin resistance was attributed to a *gadD2* missense mutation (Szendy et al., 2019). This makes it apparent that studies encompassing phenotypic and genotypic analyses of field isolates derived from different sources will lead to new understanding of the scope of molecular mechanisms and genes underlying nisin resistance in *L. monocytogenes*. We therefore investigated nisin responses variation by examining a genetically diverse collection of 356 *L. monocytogenes* strains derived from foods, food processing environment and human listeriosis cases using growth parameter-based analysis of nisin growth response phenotypes.

## Materials and Methods

### Ethics Statement

This study was performed in accordance with the principles and recommendations of the “Ordinance on laboratory animal husbandry, the production of genetically modified animals and the methods of animal experimentation; Animal Experimentation Ordinance” (SR 455.163, April 12, 2010), Swiss Federal Food Safety and Veterinary Office (FSVO/BLV). The maximum age reached by the embryos during experimentation was 5 days post fertilization (dpf) for which no license is required from the Cantonal Veterinary Office in Switzerland, since such embryos will not have yet reached the free-feeding stage. Husbandry and breeding of the adult zebrafish were performed under the supervision of Prof. Stephan Neuhauss, Institute for Molecular Life Sciences, University of Zürich, Zurich, Switzerland. All animal protocols used were in compliance with internationally recognized standards as well as with Swiss legal ethical guidelines for the use of fish in biomedical research. All the experiments were approved by the local authorities (Veterinäramt Zürich Tierhaltungsnummer 150).

### Bacterial Strains

*Listeria monocytogenes* field strains (*n* = 356) examined in this study were isolated in Switzerland (*n* = 200) and Finland (*n* = 156) from different food, animal and human-associated sources and have been previously described (Supplementary Table S1; Althaus et al., 2014; Ebner et al., 2015; Aalto-Araneda et al., 2020). The isolates belonged to 39 multilocus sequence type (MLST) clonal complexes (CC: seven loci MLST), evolutionary genetic lineages I (LI; *n* = 100) and II (LII; *n* = 256) and serotypes [1/2a (*n* = 224), 1/2b (*n* = 35), 1/2c (*n* = 32), and 4b (*n* = 65)]. Stock cultures of the strains preserved in Brain Heart Infusion (BHI) broth (Oxoid, Pratteln, Switzerland) supplemented with 20% glycerol frozen at −80°C were plated out on BHI agar (Oxoid, Pratteln, Switzerland) plates and incubated 18 h at 37°C. Single colonies from each strain were inoculated in 5 ml BHI broth and incubated 16 h at 37°C and 150 rpm. Primary cultures were sub-cultured (1:100) in BHI broth and similarly grown giving secondary stationary phase stage cultures that were subsequently used to conduct experiments described in this study unless otherwise mentioned.

### Determination of Nisin Response Phenotypes

Nisin susceptibility phenotypes were assessed using a microtiter plate based BHI broth assay. Secondary cultures from each strain were diluted in BHI (10^5^ CFU/ml) and distributed in duplicate 100 μl aliquots into a non-tissue culture treated 96 well microtiter plates (Corning Incorporated, New York, United States), which had been prefilled with 100 μl of either normal (control) or nisin-supplemented (25 ppm; 2500 IU ml^–1^; Sigma-Aldrich Co., MO, United States) BHI broth. The final nisin working concentration achieved was 12.5 ppm (500 IU ml^–1^) in line with the nisin concentration range applied in most foods and countries (Ross et al., 2002). Growth was monitored at 30-min intervals by measuring optical density (OD) at 600 nm in Synergy HT OD reader (BioTek, Lucerne, Switzerland) over 24 h at 37°C with continuous medium shaking. Strains were assessed as duplicates in three independent biological experiments.

### Estimation of Growth Curve Parameters

Growth parameter for each strain in normal and nisin supplemented BHI were determined based on growth curves generated from optical density measurements by the plate reader using the R package ‘*opm*’ as previously described (Göker, 2016; Göker et al., 2016). Briefly, the raw growth curve data of each replicate were organized into a user-defined data frame using the *head ()* function. A virtual plate with virtual wells was then created and registered using the *register_plate ()* function. The data from the data frame was loaded into the virtual plates using the *opmx ()* function. Four growth curve parameters, which included lag phase duration, growth rate, maximum growth rate and area under the curve (AUC), were estimated using the *do_aggr ()* function. The curve parameters from the three biological replicates were combined using the *opms ()* function and re-estimated using the *do_aggr ()*. Finally, the curve parameters were extracted as a data matrix using the *aggregated ()* function and exported into MS Excel using the *write.table ()* function. Among the four curve parameters, AUC was chosen as the nisin inhibition parameter for further analysis since it is affected by the other three parameters and was previously recommended for use in summarizing growth curves (Vaas et al., 2012). The percentage change in AUC (ΔPAUC) in response to nisin exposure was determined as follows: whereby ΔPAUC is percentage change of area under the curve after exposure to 12.5 ppm nisin, AUC_nisin_ is area under the curve after growth in BHI 12.5 ppm nisin and AUC_BHI_ is area under the curve after growth in normal BHI broth. Expected ranges were ΔPAUC < 100%, whereby higher values indicted more sensitivity to nisin. In instances where growth was below detectable limits then ΔPAUC = 100%.

$$\begin{array}{c} \Delta PAUC=100\left(1-\frac{AUC_{\text{nisin}}}{AUC_{\text{BHI}}}\right) \end{array}$$

### Classification of Nisin Response Phenotypes

Strains were categorized into components each with its own average growth parameter and corresponding proportion of the total strains using finite mixture models. To avoid subjective selection of the number of components, this was treated as a parameter in the likelihood enabling its estimation from the data using Non-Parametric Maximum Likelihood Estimation (NPMLE). This approach differs from classical Maximum Likelihood (ML) estimation theory because it allows the number of parameters in the likelihood to be flexible. An initial estimation of the potential number of sub-populations was made using vertex exchange method (VEM) (Schlattmann, 2009). Estimates from VEM algorithm were provided as starting values for the Expectation-Maximization (EM) algorithm, which was used to determine the final number of sub-populations or nisin response categories (Schlattmann, 2009). The appropriateness of the number of categories computed using the VEM and EM algorithms was checked using parametric bootstrap test (Schlattmann, 2009) by forward or backward elimination procedure for the number of categories (Schlattmann and Böhning, 1993). The final model from the above step was used to classify all isolates according to the final categories based on posterior probabilities which express how likely an *i*^th^ strain is to belong to category *g*, considering the observed AUC for that strain. The classification rule involves classifying isolate *i* into the nisin stress response category to which it has the highest probability of belonging (Schlattmann, 2009). VEM, EM and diagnosis concerning the number of subgroups were computed in R package ‘*CAMAN.*’

### Genomic Analysis

Isolates of the 356 *L. monocytogenes* field strains examined in this study were sequenced using Illumina HiSeq PE250 platform with an aimed coverage of 100x at the Institute for Molecular Medicine Finland (FIMM, Helsinki, Finland) and deposited under Bioproject PRJNA641855. Genomes of *L. monocytogenes* strains from serotypes 1/2a (EGDe; GCA_000196035.1 and 10403S; GCA_000168695.2), 1/2b (L2624; GCA_001027165.1 and G6054; GCA_003417835.1), 1/2c (LO28; GCA_000168675.1 and FSL-R2-0561; GCA_000168575.2) and 4b (Scott A; GCA_000212455.1 and H7858; GCA_000167155.1) and Firmicutes, namely *Staphylococcus aureus* strain MW2; GCA_000011265.1, *Bacillus subtilis* strain 168; GCA_000009045.1, *Bacillus cereus* strain ATCC 14579; GCA_000007825.1 and *Lactococcus lactis* strain Il1403; GCA_000006865.1, used as reference strains and for comparison were downloaded from the NCBI GeneBank database^1^. All genomes were annotated and compared using the Rapid Annotation Subsystem Technology (RAST) and Seed Viewer using standard settings^2^. To compare the known *L. monocytogenes* nisin response genes (Supplementary Table S3) a “one vs. all” genome comparison with the *L. monocytogenes* EGDe genome as the reference was used to align and extract the specific gene sequences from the genome assemblies in RAST. For quality control, all extracted gene sequences were blasted in NCBI blast website^3^ and the domains in the encoded proteins were checked using the NCBI conserved domain website^4^. Sequences of genes and their corresponding proteins were aligned and compared using CLC Genomics Workbench (Qiagen, Prismet, Denmark). The seven house-keeping gene based MLST profiles were extracted from the genome assemblies and the 356 *L. monocytogenes* field strains were assigned to sequence types and clonal complexes (CC) according to the scheme of the Institut Pasteur^5^. The high resolution genetic relatedness of the 356 *L. monocytogenes* field strains was examined at the clonal complex level by core genome based SNP comparisons using parsnp within the harvest suite (Treangen et al., 2014). Generated SNP matrices visualized in a heatmap using clustvis (Metsalu and Vilo, 2015) were used to select strains that were genetically closely related to the high nisin resistance and sensitivity phenotypic category strains, which were used in genome-wide sequence comparison and as positive clonal control strains in the phenotypic characterization assays. Core genome based SNPs for the 40 high nisin resistance and sensitivity category strains were also generated using parsnp. A Newick tree file was generated using harvesttools within the harvest suit and a maximum-likelihood phylogenetic tree was constructed using Figtree 1.4.4^6^.

### Phenotypic Characterization of Nisin Resistant and Sensitive Strains

Phenotypic characteristics that included response to cell envelope targeting agents, were determined in selected strains that included high nisin resistance strains harboring RsbU G77S and PBPB3 V240F mutations and high nisin sensitivity strains carrying mutations in *sigB*, *vir* and *dlt* operon genes as well as eight control strains (Supplementary File 1: Supplementary Tables S3A,B) without such mutations as confirmed through genome sequencing were included in the analyses. The lysozyme minimum inhibitory concentrations (MICs) were determined in 96 well microtiter plates filled with BHI supplemented with decreasing (10,000, 5,000, 2,500, 1,250, 625, 312, 156, 39, 19 μg/ml) lysozyme (Sigma-Aldrich Co., MO, United States) concentrations and incubated for 18 h at 37°C without shaking after which OD measurements were performed at 600 nm. The MIC for lysozyme corresponded to lowest concentration preventing growth (OD_600_ ≤ 0.1). Strains harboring *sigB* operon and pbp*B3* mutation and corresponding control strains were harvested and washed twice in PBS (Life Technologies Ltd., Paisley, United Kingdom) at 8,000 *g* for 5 min. Inocula (10^5^ CFU/ml) were exposed to 2% Triton X-100 (Sigma-Aldrich Co., MO, United States) in PBS for 6 h at 37°C while shaking (150 rpm), after which appropriate dilutions were plated on BHI agar and incubated at 37°C for 24 h. Colonies were counted and expressed as log CFU/ml. Survival rate was expressed as log CFU/ml difference before and after exposure to the 2% Triton X-100. Thirdly, the growth parameters in 0.075 μg/ml bacitracin (Sigma-Aldrich Co., MO, United States) of strains showing mutations in *virB* and *virR* genes and their control strains were determined and the ΔPAUC calculated as described above. Cytochrome c binding was determined for strains with *dlt* operon mutations and their corresponding control strains as previously described (Kang et al., 2015). Briefly, cultures grown to late exponential phase [OD 1.0 (10^9^ CFU/ml)] at 37°C were harvested by centrifugation (8,000 × *g* for 5 min), washed twice (8,000 × *g* for 5 min) with 20 mM MOPS [3-(N morpholino) propanesulfonic acid] buffer (pH 7) (Sigma-Aldrich Co., MO, United States). Cells were standardized to OD_600_ 0.25 (10^8^ CFU/ml) in the same buffer and incubated in cytochrome c (Sigma-Aldrich, St. Louis, MO, United States) solution (50 μg/ml) for 15 min at room temperature. Afterward, samples were centrifuged (13,000 *g* for 5 min) and the supernatant absorbance determined at 530 nm. Cytochrome c binding was calculated as follows: whereby OD_530_ with cells is the OD determined for MOPs with cytochrome *c* and cells whereas OD_530_ without cells is the OD determined for MOPs with cytochrome *c*, but without cells.

$$\begin{array}{c} \%Boundcytochromec=100\left(1-\frac{OD_{530}withcells}{OD_{530}withoutcells}\right) \end{array}$$

### Reverse Transcription Quantitative PCR Analysis

Reverse transcription quantitative-PCR (RT-qPCR) was performed to determine the impact of *sigB* and *vir* operon mutations on mRNA levels of SigB (*gadD3*) and VirR (*dltA* and *mprF*) dependent genes during growth under nisin stress using the primers listed in Supplementary Table S4. Exponentially growing 50 ml cultures of normal and nisin (1.25 ppm for all strains and 0.125 for the *virB* mutant and control strain) supplemented BHI cultures were harvested in RNA protect Bacteria reagent (Qiagen, Hombrechtikon, Switzerland) and resuspended in 0.5 ml RNeasy Plus Mini Kit lysis buffer (Qiagen, Hombrechtikon, Switzerland). RNA was isolated as previously described (Kropac et al., 2019), quantified using Quantus Fluorimeter (Promega, WI, United States) and quality controlled using RNA 6000 Pico Chip kit and the BioAnalyzer (Agilent Technologies, United States). Reverse transcription was carried out using 1 μg of RNA (RNA integrity number ≥ 8.0) from each sample. Real-time PCR reactions were performed on the LC 480 (Roche Molecular Diagnostics, Risch-Rotkreuz, Switzerland) instrument in 20 μl reaction volumes that contained 14 ng cDNA, 0.4 μM primers and 1X LC^R^ 480 SYBR Green I master mix (Roche Molecular Diagnostics, Penzberg, Germany). Controls in which no RT was added were included to rule out DNA contamination of the RNA samples. Real-time PCR cycling conditions were as previously described (Kropac et al., 2019). Relative cDNA quantification was performed using the Light Cycler 480 Relative Quantification Software (Roche Molecular Diagnostics). The amounts of mRNAs were normalized using 16S rRNA as a reference gene (Tasara and Stephan, 2007). Each sample was analyzed in three independent biological replicates with two technical replicates each.

### Virulence Analysis Using Zebrafish Larvae

In order to determine whether the mutations identified in the nisin tolerant and sensitive strains had an impact on virulence, two nisin tolerant *L. monocytogenes* strains, LK60/1 and LT12/1 with p*bpB3* V240F and *rsbU* G77S mutations, respectively and three nisin sensitive strains, TT82E, N12-2449 and N11-1846, with mutation in *dltA*, *virB* and *rsbU* genes, respectively, were compared with genetically close strains from the same CC (Supplementary File 1: Supplementary Tables S3A,B) but without mutations in these or the other known nisin response genes. The virulence assays were carried out using 2-day post fertilization zebrafish embryos infected through microinjection as previously described (Eshwar et al., 2017). A 100 CFU inoculum prepared in Dulbecco’s Phosphate-Buffered Saline (DPBS) was injected into the embryos’ blood stream through the caudal vein and the embryos were allowed to recover in fresh E3 medium (5 mM NaCl, 0.17 mM KCl, 0.33 mM CaCl2, and 0.33 mM MgSO4) for 15 min. Post infection the embryos were incubated at 28°C and observed for mortality under a stereomicroscope twice a day over a 3 days period. *L. monocytogenes* EGDe was included in the assay as a positive control and DPBS without bacterial suspension was included as negative control.

### Prediction of Protein 3D Structure and Structural Changes Introduced by Amino Acid Substitutions

The 3D structure of proteins with an amino acid substitution predicted to result in either a nisin hyper-resistance or hyper-sensitivity phenotype were determined using PHYRE2 (Kelley L. A. et al., 2015). Structural changes introduced by an amino acid substitution in genes of interest were predicted and modeled using the 3D online pipeline^7^ as previously described (Ittisoponpisan et al., 2019). The changes include; breakage of disulphide bonds and buried H-bonds and salt bridges, introduction of buried residue, hydrophilic residues and charges, replacement of buried charges and amino acid residues, alteration of secondary structure and cavities, switching between buried and exposed residues and charges, occurrence of the glycine in a bend, a disallowed phi/psi and clash alert. The structural changes are described in detail elsewhere (Ittisoponpisan et al., 2019).

### Data Analysis

Initially, data analyses were carried out in R Studio Version 1.1.463 using the “*ggpubr*,” “*ggplot2*,” “*data.table*” and “*scales*” packages (RStudio, Inc., Boston, United States). The AUC data, AUC in normal and nisin supplemented BHI as well that of ΔPAUC were described using means and standard deviations. Distribution of ΔPAUC Lineages, Serotypes, MLST CC, and sample sources were determined using box plots while the distribution of nisin tolerant, intermediate and sensitive strains among sample sources and the distribution of *L. monocytogenes* strains with either increased tolerance or sensitivity to nisin among MLST CC were determined using bar plots. Bar plots were also used to compare phenotypic responses to lysozyme, Triton X-100, bacitracin and binding to cytochrome *c* among respective strains with and without mutations in specific genes or loci. Differences in means (*p* ≤ 0.05) for ΔPAUC among Lineages, Serotypes, MLST CC and sample sources as well as means for phenotypic responses to lysozyme, Triton X-100, bacitracin and binding to cytochrome c among highly tolerant or sensitive nisin strains and control strains were compared either using parametric and non-parametric as appropriate. Lastly, differences in means (*p* ≤ 0.05) for *gadD3*, *dltA* and *mprF* mRNA levels nisin resistant or sensitive strains and their corresponding control strains were compared using GraphPad Prism 8 software as were the Kaplan Meier mortality analysis and statistics for experiments with zebrafish (GraphPad Software, CA, United States).

## Results

### Reduced Nisin Susceptibility Among *L. monocytogenes* Field Strains

**FIGURE 1:**
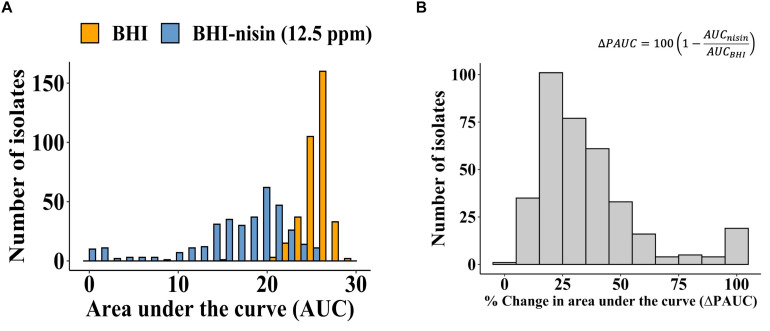
Nisin impact on growth capacity of the 356 *L. monocytogenes* field strains. **(A)** Distribution of the growth curve AUCs determined for growth in BHI and BHI-nisin. **(B)** Overall distribution of the nisin inhibition induced ΔPAUCs for the 356 *L. monocytogenes* strains displayed left skewing with a mean ΔPAUC of 35.53% indicating high nisin resistance among the examined strains.

Growth curve analysis based on area under the curve (AUC) was used for determining the nisin susceptibility phenotypes of the 356 *L. monocytogenes* filed strains that were isolated from different food associated sources, birds, food animals (cattle and sheep) and human listeriosis (Supplementary Table S1). AUCs were determined for each strain during growth in normal and nisin (12.5 ppm) supplemented BHI. An overall comparison of the AUC distributions and means between BHI and BHI-nisin grown cultures indicated that nisin inclusion in BHI caused growth inhibition as indicated by overall shift toward lower values in the growth curve AUCs and decrease in the overall AUC mean of the strains from 25.82 in BHI to 16.72 in BHI-nisin (Figure 1A). At the applied nisin concentration (12.5 ppm), growth was registered in 97% (345/356) of the examined strains, whereas in 3% (11/356) of the strains despite growth in normal BHI there was no detectable growth (OD_600_ < 0.1) in nisin supplemented BHI (Supplementary Table S1). Inspection of the generated growth curves however showed that growth capacity was strain-dependent in normal as well as nisin supplemented BHI (data not shown). In order to account for this variability, we determined strain-specific nisin inhibition induced percentage change in AUC (ΔPAUC), which was calculated by expressing growth curve AUC observed in BHI-nisin as a percentage of the uninhibited growth curve AUC observed during growth in normal BHI. The overall ΔPAUC distribution for all the strains presented in Figure 1B was skewed to the left with a mean of 35.53%, which indicates overall low susceptibility to nisin inhibition in the majority of the examined *L. monocytogenes* field strains (Figure 1B).

### Nisin Susceptibility Varies With *L. monocytogenes* Molecular Subtypes and Isolation Sources

**FIGURE 2:**
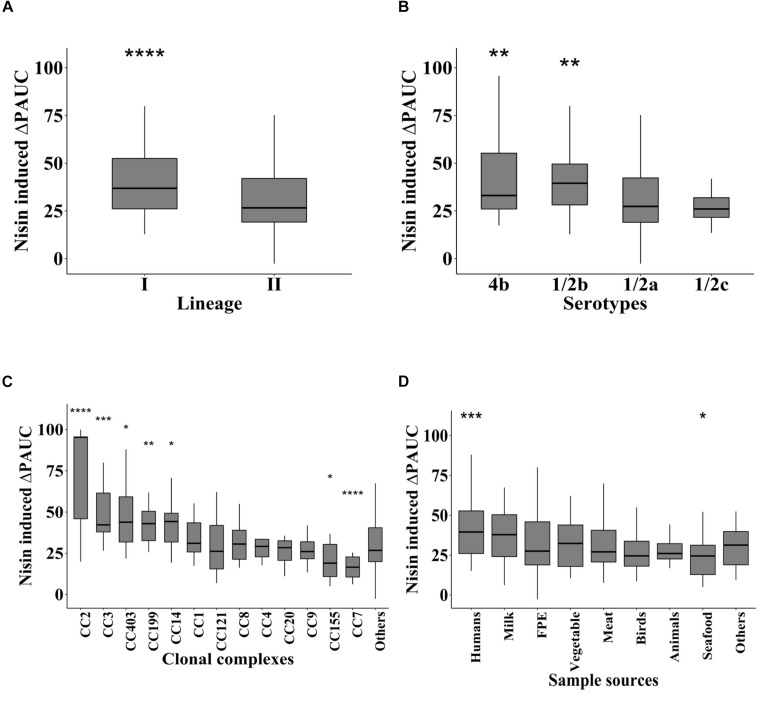
Nisin resistance variation is associated with *L. monocytogenes* molecular subtypes and isolation sources. Nisin inhibition induced ΔPAUC comparisons between *L. monocytogenes* strains grouped according to **(A)** genetic lineage, **(B)** serotype and **(C)** MLST CC and **(D)** isolation sources. Significant differences between the groups identified using one-way ANOVA and Tukey *post hoc* test pairwise comparison of the different groups. ^∗^*P* < 0.05; **P < 0.01, ***P < 0.001, ****P < 0.0001.

Comparison of nisin induced growth inhibition as judged by the ΔPAUC with respect to strain serotypes, evolutionary genetic linage and MLST CC revealed significant differences in nisin sensitivity trends. LII serotype 1/2a and 1/2c strains were significantly more resistant to nisin than the LI serotype 1/2b and 4b strains (Figures 2A,B). Strains from MLST CC7 and CC155 had significantly low nisin sensitivity whereas those from CC2, CC3, CC14, CC199, and CC403 displayed significantly higher nisin sensitivity levels compared with strains of the other CCs (Figure 2C). Sample source-based comparison of the strains showed that seafood isolates in our strain collection were more resistant (lowest ΔPAUC) whereas human derived isolates were more (highest ΔPAUC) sensitive to nisin compared to isolates from other isolation sources (Figure 2D).

### Nisin Response Phenotype Distribution Among the Examined *L. monocytogenes* Field Strains

**FIGURE 3:**
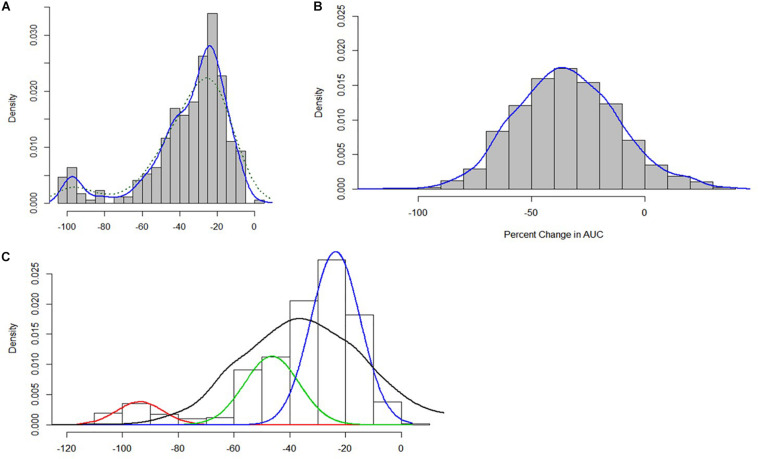
**(A)** Distribution of nisin stress response ΔPAUC values for the 356 *L. monocytogenes* strains **(B)** ΔPAUC assuming a homogeneous normal distribution **(C)** Mixture distributions underlying the ΔPAUC.

An exploration of the distribution of the ΔPAUC values associated with growth under nisin stress conditions indicated multi-modality which suggested population heterogeneity in stress response categories within our strain collection that could not be reliably described by stress category cut-offs from a single distribution (Figure 3A).

Finite mixture models using a Non-Parametric Maximum Likelihood Estimation model (Schlattmann, 2009) were used to determine the number of mixture distribution or stress sub-populations. The resulting model was then used to assign the 356 *L. monocytogenes* strains into statistically distinct (*p* < 0.05) nisin response phenotypic groups based on their ΔPAUC values. A homogeneous normal distribution assumed an average ΔPAUC of 35.48 ± 22 (Figure 3B). The finite mixture model consisted of the three-component model:

$$\begin{array}{c} \mu ∼\left(\begin{array}{ccc} 94 & 47 & 24\\ 0.08 & 0.26 & 0.66 \end{array}\right) \end{array}$$

This model consisted of a weighed sum of normal distributions:

$$\begin{array}{c} Y∼0.08N\left(94,77.4\right)+0.26N\left(47,77.4\right)+0.66\left(24,77.4\right)\left(\text{Figure}3\text{C}\right) \end{array}$$

**FIGURE 4:**
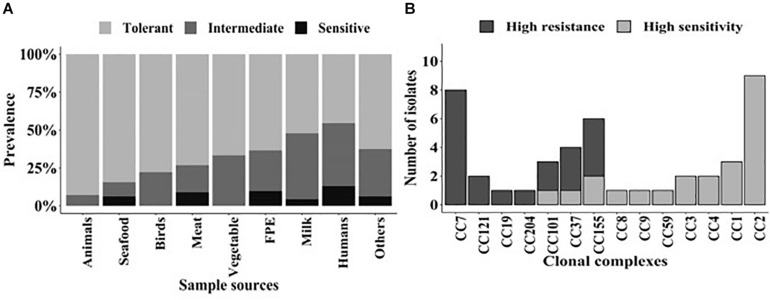
**(A)** Distribution of the 356 *L. monocytogenes* field strains classified into the resistant (−2.4% ≤ ΔPAUC ≤ 38.2%), intermediate (38.7% ≤ ΔPAUC ≤ 70.7%), and sensitive (74.7% ≤ ΔPAUC ≤ 100%) nisin response phenotypic categories based on sample sources. Strains were assigned into the three nisin response phenotypic categories based on Non-Parametric Maximum Likelihood Estimation models. **(B)** Distribution of the MLST CCs detected among the high nisin resistance (ΔPAUC < 10%) and sensitivity (ΔPAUC > 90%) category strains corresponding to the upper (resistant) and lower (sensitive) 10% of the ΔPAUC distribution, respectively.

**FIGURE 5:**
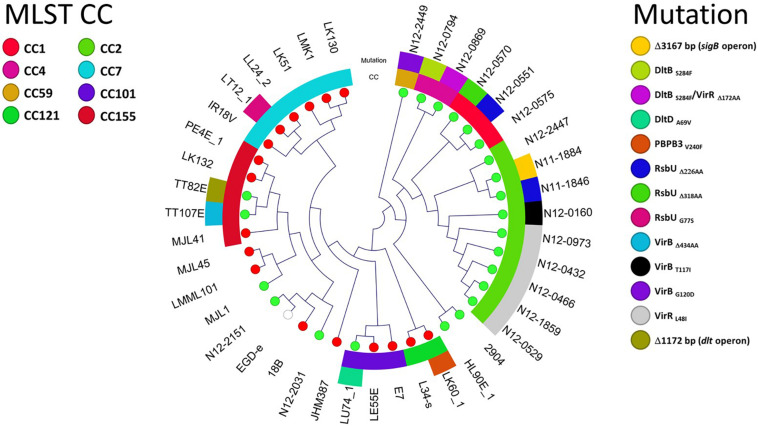
Overview of 40 *L. monocytogenes* strains showing the highest nisin resistance (ΔPAUC < 10%; 17 strains) and sensitivity (ΔPAUC > 90%; 23 strains). Cladogram showing the phylogenetic relationship of the strains based on core genome SNPs. The MLST CCs and nisin response gene mutations detected among the nisin resistant (red leaf nodes) and sensitive (green leaf nodes) strains are also highlighted.

Of these three components, the first component was the category of strains with the highest mean ΔPAUC of 94 and represented 8% of the isolates. This population could be considered as nisin stress sensitive strains. The second component comprised 26% of the strains with an intermediate mean ΔPAUC of 47, and these could be considered as intermediate strains. The third component consisted of strains with the least mean ΔPAUC of 24 and comprised the majority (66%) of the strains that could be considered as nisin stress resistant. Based on this, the strains were thus assigned to resistant (−2.4% ≤ ΔPAUC ≤ 38.2%), intermediate (38.7% ≤ ΔPAUC ≤ 70.7%), and sensitive (74.7% ≤ ΔPAUC ≤ 100%) groups with respect to their nisin sensitivity (Supplementary Table S1). Notably this categorization also reflected the ΔPAUC distribution observations already described above showing that most of the examined field strains were poorly inhibited by nisin at 12.5 ppM (Figure 1B). Based on sample sources we found that the prevalence of nisin resistant phenotypes was highest among food animal (13/14; 93%) and seafood (27/32; 84%) isolates whereas human isolates showed the highest prevalence of sensitive strains (10/77; 13%) (Figure 4A). Next we selected and focused the rest of our study on the group of highest nisin resistance and sensitivity among the strains that corresponds to the upper (highest nisin resistance) and lower (highest nisin sensitivity) 10% of the nisin response ΔPAUC distribution. All in all there were seventeen nisin resistant (ΔPAUC < 10%) and twenty-three nisin sensitive (ΔPAUC > 90%) strains from our study meeting these criteria (Supplementary Table S2). An overview of MLST CC distribution within this category of strains presented in Figure 4B shows that the majority of high nisin resistance and sensitivity strains belonged to MLST CC7 and CC2, respectively. CC121 and CC155 had two strains each that showed increased high nisin resistance while CC1, CC3 and CC4 had at least two strains each that showed high nisin sensitivity (Figure 4B). The high nisin resistance and sensitivity category of strains (40 strains) was further subjected to whole genome sequence analysis focusing on genetic variations associated with genetic elements that are currently known to play a roles in nisin responses. Figure 5 shows a core genome based SNP phylogeny of these 40 strains with their corresponding nisin response phenotypes, MLST CC affiliation and strain-specific genetic mutations in known nisin response genes uncovered from comparative analysis of their genome sequence analysis with the rest of our strain collection and reference *L. monocytogenes* strains.

### Natural PBPB3 V240F and RsbU G77S Mutations Are Associated With Increased Nisin Resistance

**FIGURE 6:**
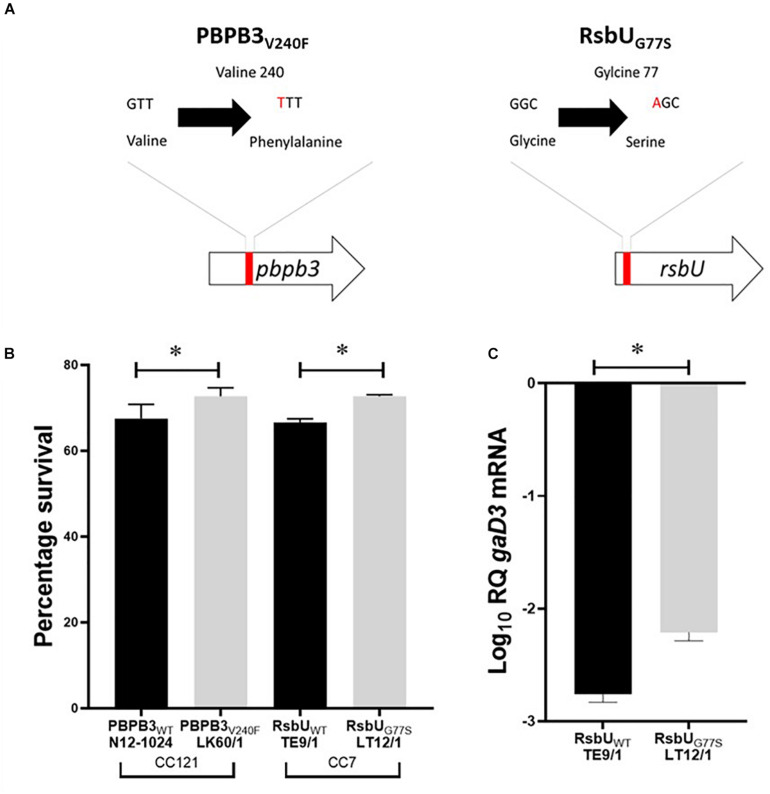
**(A)** Schematic representation of the PBPB3_V240F_ and RsbU_G77S_ mutations detected in nisin resistant *L. monocytogenes* field strains LK60/1 and LT12/1. **(B)** Triton X-100 stress survival analysis **(C)** RT-PCR quantification of *gadD3* mRNA levels in exponentially growing *L. monocytogenes* cells cultivated in under nisin stress in BHI at 37°C. Data represents the mean and standard deviation from three independent biological experiments. *P < 0.05 significant difference between the strains based on pairwise *t*-test comparison for independent samples.

Genome-wide sequence analysis and comparison of our *L. monocytogenes* field strain collection revealed strain specific point mutations in *pbpb3* (*lmo0441*) and *rsbU* genes of two of the seventeen high nisin resistance category (ΔPAUC < 10%) strains, LK60/1 and LT12/1 (Figure 5 and Table 1). These two mutations caused PBPB3 (PBPB3_V240F_) and RsbU (RsbU_G77S_) amino acid substitutions. Such mutations were unique to the two high nisin resistance strains being absent from genomes of the rest of our field strain collection as well as genomes of the reference and other *L. monocytogenes* strains deposited in public data bases. *L. monocytogenes* LK60/1 harbouring the PBPB3_V240F_ is a MLST CC121 strain. It has non-conserved amino acid change due to a 718 G > T point mutation within the *pbpB3* gene that changes the encoded amino acid at position 240 of the PBPB3 protein from valine (GTT) to phenylalanine (TTT) (Figure 6A and Table 1). The V240 residue replaced in this mutation is otherwise conserved in *L. monocytogenes*, *B. subtilis* and *B. cereus* and *S. aureus* PBPB3 proteins (Supplementary Figure S1). *L. monocytogenes* LT12/1 carrying the RsbU_G77S_ mutation is a MLST CC7 strain that due to an *rsbU* 229 G > A point mutation acquired a GGC to an AGC codon change substituting G77 for a serine in the RsbU protein (Figure 6A and Table 1). The substituted G77 residue is conserved in *L. monocytogenes*, *B. subtilis* and *S. aureus* RsbU proteins (Supplementary Figure S2).

**TABLE 1:** Natural nisin resistance gene mutations associated with high nisin resistance (ΔPAUC < 10%) and sensitivity (ΔPAUC > 90%) *L. monocytogenes* field strains.

Strain	MLST CC	ΔPAUC^1^	Nisin response phenotype	Mutation
LK60/1	CC121	6.94	R	718G > T *pbpB3* (*lmo0441*) mutation causing PBPB3_V240F_ substitution
LT12/1	CC7	7.64	R	229G > A *rsbU* mutation causing RsbU_G77S_ substitution
N12-055**1**	CC1	95.77	S	295A deletion in the *rsbU* gene causing frameshift and a L109 (TTA) → Stop (TAG) codon change causing an RsbU_Δ226AA_ truncation
N11-1846	CC2	100	S	322A deletion in the *rsbU* gene causing frameshift and a L109 (TTA) → Stop (TAG) codon change causing a RsbU_Δ226AA_ truncation
N12-0570	CC1	95.78	S	32A deletion in the *rsbU* gene causing frameshift and a L16 (TTA) → Stop (TAG) codon change causing a RsbU_Δ318AA_ truncation
N11-1884	CC2	100	S	3167 bp *sigB* operon deletion of the *rsbR*, *rsb*S, *rsbT*, *rsbU*, and part of the *rsbV* gene
N12-0869	CC4	100	S	103 bp (91G-193A) *virR* deletion that causes an open reading frame shift and an D54 (AAT) →Stop (TAA) codon change causing a VirR_Δ172AA_ truncation. A 851C > T *dltB* mutation causing DltB_S284F_ substitution
N12-0466	CC2	95.37	S	142T > A *virR* mutation that induces a VirR_L48I_ amino acid substitution mutation
N12-0973	CC2	95.49	S	142T > A *virR* mutation that induces a VirR_L48I_ amino acid substitution mutation
N12-0432	CC2	95.54	S	142T > A *virR* mutation that induces a VirR_L48I_ amino acid substitution mutation
N12-0529	CC2	95.57	S	142T > A *virR* mutation that induces a VirR_L48I_ amino acid substitution mutation
N12-1859	CC2	95.79	S	142T > A *virR* mutation that induces a VirR_L48I_ amino acid substitution mutation
TT107E	CC155	100	S	710 G > A *virB* mutation that causes a W236 (TGG) →Stop (TAG) codon change causing a VirB_Δ434AA_ truncation
N12-2449	CC59	100	S	392G > A *virB* mutation causing a VirB_G120D_ amino acid substitution
N12-0160	CC2	100	S	383C > T *virB* mutation causing a VirBT_117I_ amino acid substitution
TT82E	CC155	100	S	1172 bp deletion of the *dlt* operon removing the *dltA* operon promoter, *lmo0975*, hypothetical protein gene, and the first 67 codons of *dltA*.
N12-0794	CC4	100	S	851C > T *dltB* mutation causing DltB_S284F_ amino acid substitution
LU74/1	CC101	91.54	S	506C > T *dltD* causing a DltD_A69V_ amino acid substitution

*^1^ΔPAUC; Nisin inhibition induced percentage change in area under the curve.*

**FIGURE 7:**
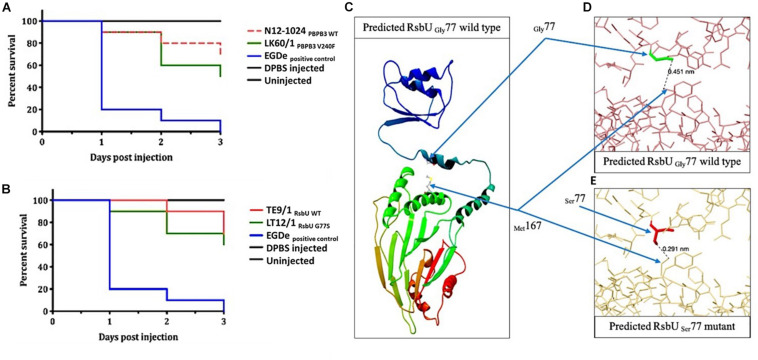
Virulence evaluation of the high nisin resistance *L. monocytogenes* field strains harbouring the PBPB3_V240F_ and RsbU_G77S_ mutations and theoretical structural modeling prediction analysis of the RsbU_G77S_ mutant protein. Kaplan–Meier plots depicting the survival trends of zebrafish embryos (*n* = 30 per *L. monocytogenes* strain) infected (100 CFU per embryo) with the high nisin resistance mutant strains **(A)** LK60/1 (PBPB3_V240F_; CC121) and **(B)** LT12/1 (RsbU_G77S_; CC7) and their respective clonal control strains, N12-1024 (CC121) and TE9/1 (CC7), without mutation. Data are based on three independent biological experiments. Survival rate and trends at 48 and 72 h post infection were significantly lower in LK60/1 (PBPB3_V240F_) and LT12/1 (RsbU_G77S_) infected zebrafish embryos infected compared to their respective control strains N12-1024 and TE9/1 (*P* < 0.05). **(C)** Gly^77^ and Met^167^ in wild type RsbU as predicted in Phyre2. **(D)** RsbU Gly^77^ wild type showing the distance (nm = 0.451) between Gly^77^ and Met^167^ as predicted by Missense3D. **(E)** Missense3D-predicted RsbU Ser^77^ mutant showing the distance (nm = 0.291) between Ser^77^ and Met^167^, and the possible cause for 85.5 Å^3 collapse in cavity volume after G77S substitution. The figures were generated in **(C)** CLC Genomics Workbench and **(D,E)** Missense3D online server.

Since intrinsic nisin resistance mechanisms include cell envelope based protection we reasoned that phenotypic consequences of the PBPB3_V240F_ and RsbU_G77S_ mutations might also manifest through changes in general cell envelope stress resistance in the mutated nisin resistant strains. To examine this, we compared resistance to lysozyme and Triton X-100 (detergent) associated envelope stress between LK60/1 and LT12/1 strains carrying PBPB3_V240F_ and RsbU_G77S_ variants, with their corresponding non-mutated clonal positive control strains *L. monocytogenes* N12-1024 and TE9/1, respectively. Strain LK60/1 harboring PBPB3_V240F_ displayed higher Triton X-100 but similar lysozyme resistance to its clonal control strain N12-1024 without such a PBPB3 mutation (Figure 6B and Supplementary Figure S3). *L. monocytogenes* LT12/1 harboring RsbU_G77S_ showed higher Triton X-100 as well as lysozyme resistance when compared its clonal positive control possessing a non-mutated RsbU protein *L. monocytogenes* TE9/1 (Figure 6B and Supplementary Figure S3). Thus, these two high nisin resistance category strains harboring PBPB3_V240F_ and RsbU_G77S_ also exhibit increased cell envelope stress resistance compared to those with non-mutated protein variants. The impact of the RsbU_G77S_ mutation in *L. monocytogenes* LT12/1 on SigB activation was indirectly examined by comparing its levels of the SigB-dependent *gadD3* mRNA to those of its clonal control strain *L. monocytogenes* TE9/1. We found that exponentially growing LT12/1 cells cultivated under nisin stress contained higher *gadD3* mRNA abundance than clonal control strain TE9/1 cells (Figure 6C). These observations thus also confirmed higher SigB activation under nisin stress growth conditions in strain LT12/1, which possesses the RsbU_G77S_ protein variant and exhibits high nisin resistance. We hypothesized that besides conferring increased nisin resistance the PBPB3_V240F_ and RsbU_G77S_ variants could also have consequences on *L. monocytogenes* resistance in the context of host infection and virulence potential. To assess this, the virulence capacities of the LK60/1 and LT12/1 strains was evaluated in a zebra fish embryo based infection model. Comparing mortality induction, we found significantly higher mortality in zebra fish embryos infected with the LK60/1 (PBPB3_V240F_) and LT12/1 (RsbU_G77S)_ strains when compared to those infected with their corresponding clonal control strains N12-1024 and TE9/1, without such PBPB3 and RsbU protein mutations, respectively (Figures 7A,B). We also examined for possible consequences of these non-synonymous amino acid changes in the predicted structures of the resulting PBPB3_V240F_ and RsbU_G77S_ protein variants. Theoretical structural modeling programs Missense 3D and Phyre2 were used and the predicted structures of the PBPB3_V240F_ and RsbU_G77S_ variants and their non mutated proteins were compared. Although the PBPB3_V240F_ mutation is localized within the structurally conserved PBPB3 allosteric domain, there were no structural impacts predicted from this amino acid change suggesting this mutation does not have localized structural consequences on the resulting PBPB3 protein (Supplementary Figure S4). On the other hand, there were significant protein structural alterations predicted for the RsbU_G77S_ variant. More specifically while the G77 (RSA 5.9%) residue is predicted to be buried in the normal RsbU protein structure the substituting S77 residue of the RsbU_G77S_ protein variant was predicted to be exposed (RSA 12.3%) causing an 85.5 Å^3^ volume cavity contraction in the RsbU_G77S_ protein variant compared to the normal RsbU protein (Figures 7C–E). These predicted structural alterations might thus have possible consequences for RsbU_G77S_ functions thereby altering the nisin response phenotypes in *L. monocytogenes* strain LT12/1 harboring this mutation. The PBPB3_V240F_ variant on the other hand appears to be associated with no structural perturbations. Such structural predictions and their consequences will however need experimental confirmation through future studies.

### Natural Mutations in *dlt, vir*, and *sigB* Operons Are Associated With Increased Nisin Sensitivity

**FIGURE 8:**
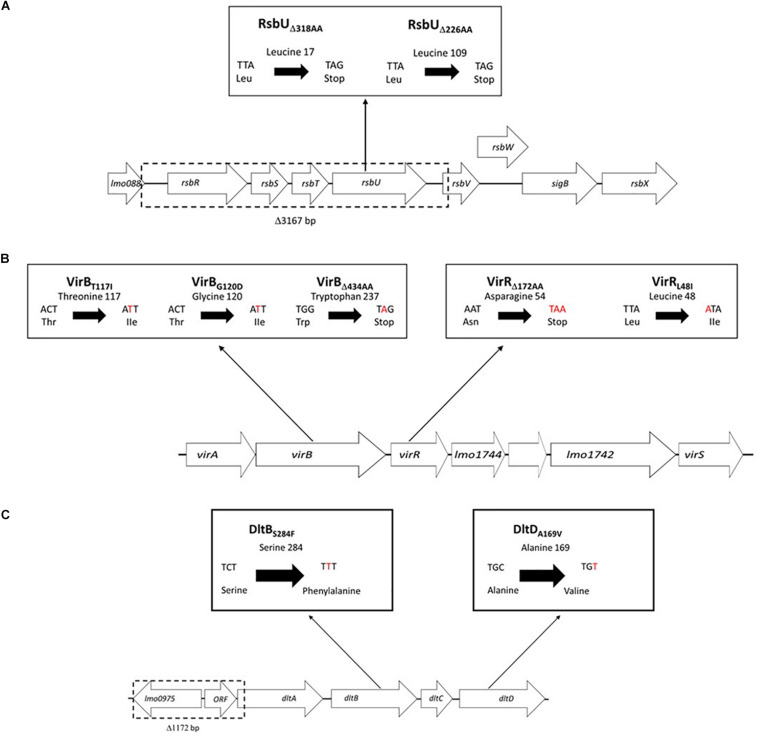
Schematic representations of the *sigB*, *vir*, and *dlt* operon associated mutations uncovered among the nisin sensitive *L. monocytogenes* field strains from our study. **(A)** In the *sigB* operon single base deletions causing RsbU truncations (RsbU_Δ__318AA_ and RsbU_Δ__226AA_) and a 31367 bp deletion (causing loss of *rsbR*, *rsbS, and rsbT* genes and part of *rsbV*) were detected. **(B)** In the *vir* operon point mutations causing amino acid substitutions and single base deletion truncated proteins were detected in the *virR* and *virB* genes. **(C)** In the *dlt* operon a 1172 bp deletion (causing loss of *lm00975*, predicted hypothetical protein ORF and *dltA* N-terminus) as well as point mutations causing amino acid substitutions in *dltB* and *dltD* genes were detected.

Whole genome sequence analysis also uncovered several strain specific mutations that only involved *sigB*, *vir*, and *dlt* operon genes among strains assigned to the high nisin sensitivity category (ΔPAUC > 90%) in our study (Figure 5 and Table 1). These mutations occurred in seventeen of the twenty three nisin sensitive strains but were absent from the rest of our field strain collection as well as the genomes of *L. monocytogenes* reference strains. In four of the strains, mutations involved genes of the *sigB* operon (Figure 8A). A 3167 bp deletion was detected in an MLST CC2 strain *L. monocytogenes* N11-1884 that causes loss of *rsbR*, *rsbS* and *rsbT* genes, as well as part of the *rsbV* gene including the protein translational start codon. A second *sigB* operon associated mutation involved an *rsbU* single base deletion (32A) in an MLST CC1 strain *L. monocytogenes* N12-0570 (Supplementary Figure S5). This deletion mutation generates a translation reading frameshift resulting in a L16 (TTA) → Stop (TAG) codon change generating an RsbU_Δ318AA_ truncation. Two nisin sensitive MLST CC2 strains, *L. monocytogenes* N12-0551 and N11-1846, affected in the *sigB* operon carried 295A and 322A single base *rsbU* deletions, respectively (Supplementary Figure S6), that in both cases induces RsbU open reading frame shifts resulting in identical premature stop codons due to a L109 (TTA) → Stop (TAG) codon change resulting in an RsbU_Δ226__AA_ truncation.

Nine of the nisin sensitive strains contained *vir* operon associated mutations located in *virR* and *virB* genes (Figure 5 and Table 1). Six nisin sensitive MLST CC2 were mutated in the *virR* gene. *L. monocytogenes* N12-0869 strain had a 103 bp (91G-193A) *virR* deletion that causes an open reading frame shift and an D54 (AAT) →Stop (TAA) codon change resulting in a VirR_Δ172AA_ truncated protein (Figure 8B). In addition, this strain also had a DltB_S284F_ amino acid substitution mutation as described below. The other five MLST CC2 nisin sensitive strains all carried an identical 142T > A *virR* mutation that induces a conserved VirR_L48I_ amino acid substitution mutation (Figure 8B). A similar amino acid substitution was also found upon inspection of genome sequences of four other *L. monocytogenes* strains (genome accession numbers: AAARPI010000014.1, AAASCZ010000015.1, AANDNJ010000012.1, and AANDMI010000029.1) deposited in the NCBI data base (Supplementary Figure S7).

**FIGURE 9:**
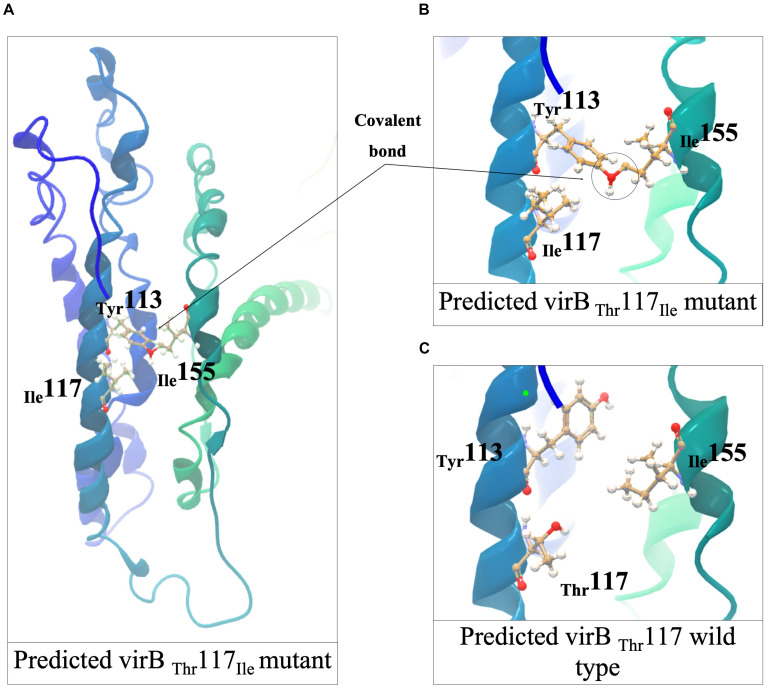
Structural predictions and analyses of the *L. monocytogenes* VirB _I117T_ mutant protein. **(A,B)** The formation of a covalent bond between T^132^ and I^155^ in opposite a-helixes in VirB_I117T_ mutant predicted using Phyre2. **(C)** Missense3D-predicted T117I (VirB wild type) compliment showed the covalent bond in T^113^ and I^155^ could be broken after I117T complementation. The figures were generated in CLC Genomics Workbench.

Meanwhile, three nisin sensitive strains all carried *virB* gene associated mutations (Figure 5 and Table 1). *L. monocytogenes* TT107E an MLST CC155 strain had a 710 G > A *virB* mutation that causes W237 (TGG) →Stop (TAG) codon change leading to a VirB_Δ434AA_ truncated protein (Figure 8B). The *virB* gene of an MLST CC59 strain (N12-2449) contained a 359G > A mutation predicted to cause a non-conserved VirB_G120D_ amino acid substitution (Figure 8B). The G120 residue substituted through this mutation is conserved in *L. monocytogenes*, *S. aureus*, *B. subtilis*, and *L. lactis* VirB proteins (Supplementary Figure S8). *L. monocytogenes* N12-0160, a nisin sensitive MLST CC2 strain contained a 383C > T *virB* mutation leading to a non-conserved VirB_T117I_ amino acid substitution (Figure 8B). This mutation is predicted to create an additional covalent bond between the opposite a-helixes of Y113 and I155 residues in VirB_T117I_ variant, which is otherwise absent in the normal VirB protein based on theoretical structural protein modeling prediction (Figure 9).

In four of the high nisin sensitivity category strains, *dlt* operon associated mutations were detected (Figure 5 and Table 1). One strain derived from CC155, *L. monocytogenes* TT82E contained a 1172 bp deletion causing *dlt* operon promoter loss, and deletion of *lmo0975* (encoding Ribose 5-phosphate isomerase A), a predicted hypothetical protein open reading frame, and the first 67 codons including the translation start codon of the *dltA* gene (Figure 8C). There was an 851C > T *dltB* gene mutation causing a non-conserved DltBS_284F_ amino acid substitution detected within the genomes of two MLST CC4 strains (N12-0794 and N12-0869) including the strain *L. monocytogenes* N12-0869, which also contained the VirR_Δ172AA_ truncation mutation described above (Figure 8C). The S284 residue substituted in this mutation is found within an LSFWFRD amino acid sequence region showing high 100% sequence identity among *L. monocytogenes*, *B. cereus*, *B. subtilis*, *S. aureus*, and *L. lactis* DltB proteins (Supplementary Figure S9). The last *dlt* operon mutation detected occurred in the genome of a CC101 high nisin sensitivity strain *L. monocytogenes* LU74/1, which had a 506C > T *dltD* mutation causing a conserved DltD_A169V_ amino acid substitution (Supplementary Figure S10).

**FIGURE 10:**
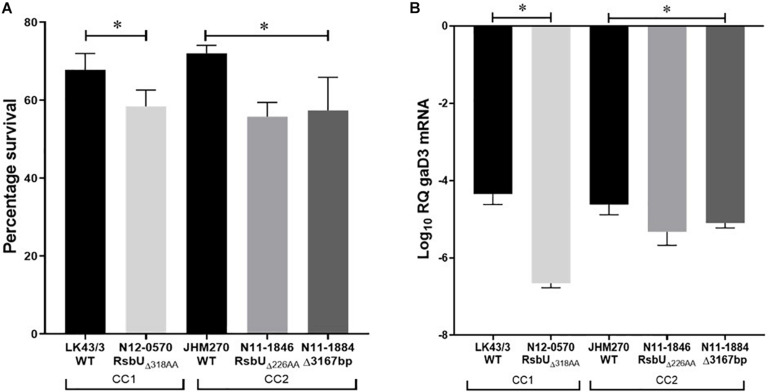
Phenotypic evaluation of high nisin sensitivity *L. monocytogenes* strains with natural *sigB* operon mutations. **(A)** Survival of Triton X-100 exposure and **(B)** *gadD3* mRNA quantification high nisin sensitivity *sigB* operon mutant strains (N11-1846, N11-1884, and N12-0570) compared with control strains (JHM270 and LK43/3) of the same MLST CC with an intact *sigB* operon. ^∗^*P* > 0.05 significant differences that were identified using one-way ANOVA and Tukey *post hoc* (groups with more than two strains) pairwise comparison of the strains.

**FIGURE 11:**
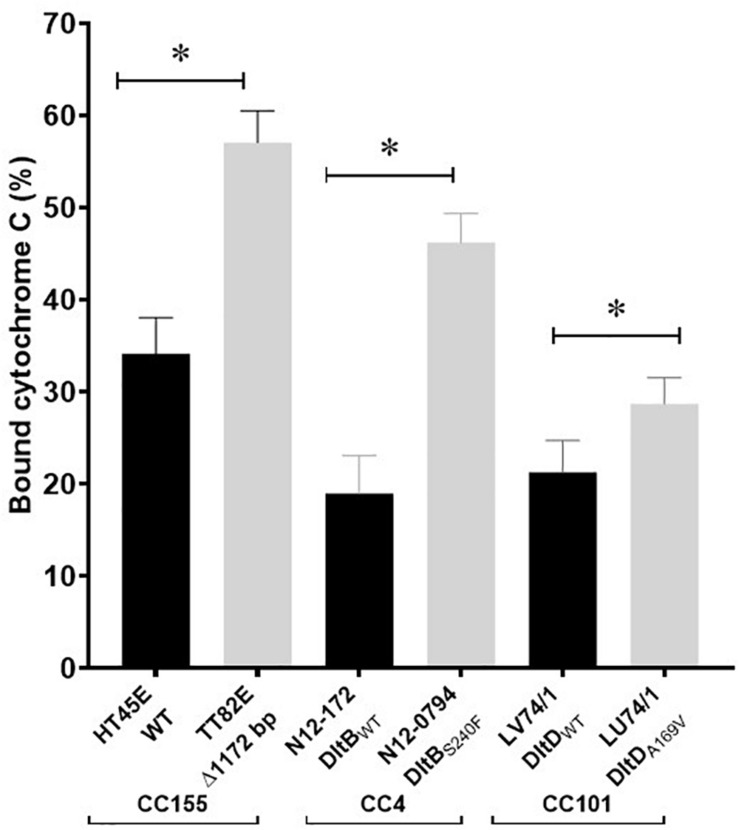
Strains with natural dlt operon mutations have an increased cell surface negative charge consistent with impaired Dlt system function. Cytochrome c binding comparison of high nisin sensitivity *dlt* operon mutant strains TT82E, N12-0794, and LU74/1 and their corresponding non-mutated clonal control strains (HT45E, N12-1772, and LL66/3). *P < 0.05 significant difference between the strains identified using the pairwise *t*-test comparison for independent samples.

**FIGURE 12:**
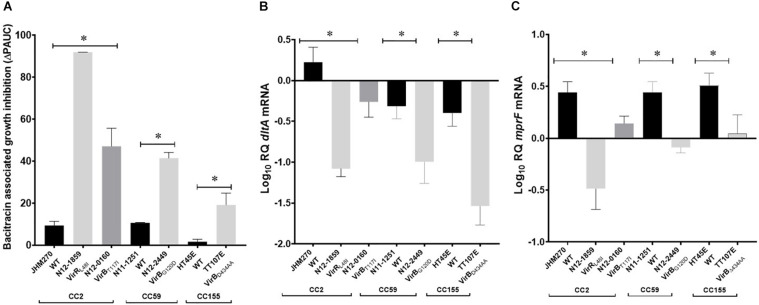
Phenotypic evaluation of high nisin sensitivity *L. monocytogenes* field strains with natural *vir* operon mutations. **(A)** Bacitracin sensitivity comparison based on bacitracin induced growth inhibition (ΔPAUC) and quantification of VirR dependent *dltA* **(B)** and *mprF* **(C)** mRNAs in high nisin sensitivity *vir* operon mutant strains (N12-1859, N12-0160, N12-2449, and LK132) compared with corresponding clonal control strains (JHM270, N11-1251, and HT45E) without *vir* operon mutations. Data presented is based on three independent biological experiments. *P > 0.05 significant differences that were identified using one-way ANOVA and Tukey *post hoc* (groups with more than two strains) pairwise comparison of the strains.

**FIGURE 13:**
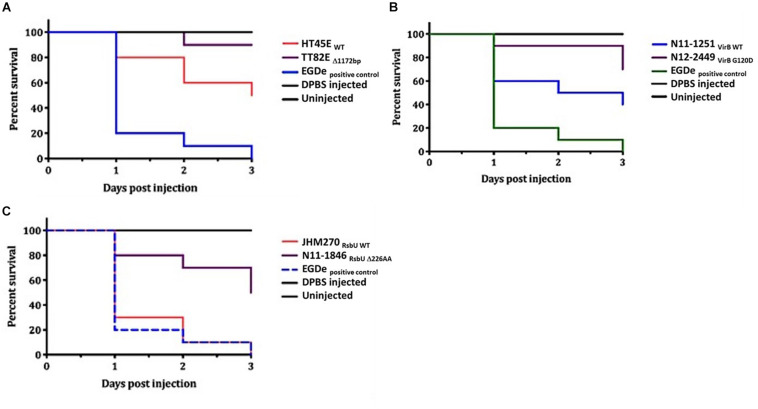
Virulence evaluation of high nisin sensitivity *L. monocytogenes* field strains harboring *dltA*, *virB*, and *rsbU* mutations using the zebrafish embryo-based infection model. Kaplan-Meier plots depicting the survivals trends of zebrafish embryos infected with strains **(A)** TT82E (deletion of entire *dlt* operon promoter and the first 67 N-terminus amino acids in DltA), **(B)** N12-2449 (VirB_G1__20__D_) and **(C)** N11-1846 (RsbU_D__226_) compared to corresponding control strains from same MLST CCs but without mutations **(A)** HT45E, **(B)** N11-1251, JHM270. Data presented is based on three independent biological experiments. Survival curves and trends were significantly higher in zebrafish embryos infected with the high nisin sensitivity strains *dltA*, *virB* and *rsbU* mutations compared to un mutated respective clonal control strains at 24, 48, and 72 hpi (*p* < 0.05).

A selection of these nisin sensitive strains described above were subjected to phenotypic characterization as an indirect way of assessing the functional impact of these natural mutations within the genetic backgrounds of the affected strains. In agreement with compromised SigB dependent stress responses, all four of nisin sensitive *sigB* operon mutant strains (N12-0551, N12-0570, N11-1846, and N11-1884) described above were also impaired in cell envelope stress resistance ability displaying increased sensitivity to Triton X-100 and lysozyme in comparison to their corresponding clonal control strains (LK43/3 and JHM270) with an intact *sigB* operon (Figure 10A and Supplementary Figure S11). In an indirect assessment of SigB activity, these *sigB* operon mutant strains were also found to express significantly lower amounts of the SigB-dependent gene *gadD3* mRNA than corresponding unmutated CC control strains (Figure 10B). The nisin sensitive *dlt* operon mutants were assessed for evidence of the phenotypic functional impairment of their Dlt systems. As loss of function mutations within this operon are expected to increase the overall cell surface anionic charge, the cytochrome c binding capacity between the nisin sensitivity *dlt* operon mutant strains and corresponding clonal positive control strains with an intact *dlt* operon were compared. All three high nisin sensitive *dlt* operon mutant strains (TT82E, N12-0794, and LU74/1) examined bound more cytochrome c than their corresponding clonal control strains (HT45E, N12-1722 and LV74/1) without mutations (Figure 11). Our observations thus indicated increased overall cell surface anionic charges, consistent with functionally impaired Dlt system and thus explaining the increased nisin sensitivity observed in these natural *dlt* operon mutants. Functional impairment of the *vir* operon is expected to increase sensitivity to cell envelope targeting agents and reduce the expression of VirR dependent genes. To validate such phenotypic impacts in the nisin sensitive natural *vir* operon mutant strains of our study we examined their sensitivity to bacitracin induced cell envelope stress and the expression of the VirR dependent *dltA* and *mprF* mRNAs in these strains. Consistent with VirR functional impairment all three *vir* operon mutant strains (N12-1859, N12-0160, N12-2449, and LK132) were also more sensitive to bacitracin and produced significantly less *dltA* and *mprF* mRNAs under nisin stress than their respective clonal positive control strains (JHM270, N11-1251 and HT45E) without mutation (Figure 12). Mutations affecting *dlt*, *vir* and *sigB* operon genes can, besides altering nisin sensitivity, be also expected to impact virulence potential in the affected strains. To validate such possible virulence impacts in the natural nisin sensitive mutant strains that are affected through the *dlt* operon deletion (TT82E with a 1172 bp deletion *dlt* operon deletion) as well as those with RsbU truncation (N11-1846) and VirB_G120D_ (N12-2449) substitution were examined using a zebra fish embryo based infection model for virulence. A comparison of the mortality rates observed upon infection of the zebra fish embryos established that all three nisin sensitive strains harbouring these mutations were significantly less virulent relative to their respective clonal control strains (HT45E, N12-2449 and JHM270) without mutations (Figure 13). Our observations thus indicated that nisin sensitive *L. monocytogenes* field strains harbor natural mutations that do not only affect nisin tolerance but can also have consequences for their host virulence potential.

## Discussion

In this study the nisin response phenotypes in 356 *L. monocytogenes* field strains isolated from various sources including food products, food-processing environments, animals and human listeriosis patients were determined through growth curve parameter-based analysis. Growth assessment was conducted at 12.5 ppm nisin corresponding to 500 IU (Younes et al., 2017), one of concentration levels currently recommended for use in food (Ross et al., 2002). Notably most of the field strains examined here were capable of efficient growth at this level of nisin concentration. Based on the strain specific nisin inhibition induced percentage change in area growth curve (ΔPAUC) most (66%) of the examined strains were nisin resistant at 12.5 ppm. In this regard our observations were similar to a previous study showing that about a third of the 282 field strains tested were still capable of significant growth at 400 IU (10 ppm) nisin in liquid media (Szendy et al., 2019). Ninety-nine percent of 381 field strains were also able to grow at 500 IU (12.5 ppm) nisin on solid media (Mota-Meira et al., 2000). Previously different studies have also found varying responses to nisin among different strains of *L. monocytogenes* with growth normally occurring below 50 ppm nisin (Benkerroum and Sandine, 1988; Ukuku and Shelef, 1997; Rasch and Knøchel, 1998; Iancu et al., 2012), while some strains exhibit growth up to 100 ppm nisin (Begley et al., 2010; Bergholz et al., 2013; Malekmohammadi et al., 2017). Differences between our present classification and those of previous studies arise from differences in the assay approaches used. While the previous studies mostly determined the MICs of *L. monocytogenes* strains or response to varying nisin concentrations (Benkerroum and Sandine, 1988; Ukuku and Shelef, 1997; Rasch and Knøchel, 1998; Iancu et al., 2012), we determined strain nisin response phenotypes at a single concentration, 12.5 ppm nisin, but taking into consideration the impact of nisin inhibition on AUC parameters of the growth curves. Our approach thus quantified a strain’s nisin tolerance based on overall growth capacity in BHI at 12.5 ppm nisin, assigning the strains to “resistant,” “intermediate,” and “sensitive” categories. Strain-specific percentage change in AUC (ΔPAUC) induced by nisin exposure were determined and compared. Despite the different study approaches, the ΔPAUC in our study like the previous nisin response studies shows an existing natural variation in nisin susceptibility among *L. monocytogenes* strains (Benkerroum and Sandine, 1988; Ukuku and Shelef, 1997; Rasch and Knøchel, 1998; Iancu et al., 2012).

Previously others showed that clinical isolates were more sensitive to nisin compared to isolates from food sources (Buncic et al., 2001). In a similar observation we found in our study that strains derived from human clinical cases displayed higher nisin sensitivity compared to strains from food and food processing environments. The frequent use of nisin in food processing as a food preservative and the presence of the nisin producing *L. lactis* strains, in foods (Cotter et al., 2005), means nisin exposure of *L. monocytogenes* occurs frequently in food associated environments. This can thus positively select for nisin tolerant *L. monocytogenes* strains leading to higher nisin resistance observed among isolates from food products and food processing environments. Indeed, mutations leading to increased nisin resistance have been demonstrated previously and reported to occur at high rates among *L. monocytogenes* strains (Harris et al., 1991; Xintian and Daeschel, 1993; Gravesen et al., 2002; Wu et al., 2017), as an influence of the environmental factors encountered in the food matrix and processing environment including salt, acidity and cold temperature (Van Schaik et al., 1999; Bouttefroy and Millière, 2000; Cotter et al., 2005).

Genetic Lineage II strains were found to be more tolerant to nisin than those from Lineage I, in agreement with previous reports (Buncic et al., 2001). Lineage differences also extended to serotype and CC level. Nisin tolerance trends of serotypes 1/2a and 1/2c > 1/2b > 4b were also observed in agreement with previous studies showing that serotype 1/2a and 4b strains were more tolerant and sensitive to nisin, respectively (Buncic et al., 2001; Katla et al., 2003; Szendy et al., 2019). Comparing the strains at the MLST CC level we found that CC7 strains displayed highest nisin resistance whereas CC2 and CC3 strains showed the lowest nisin resistance levels similar to previous observations (Malekmohammadi et al., 2017). In addition, our study brought new insights regarding the nisin response phenotypes among strains of other rarely studied CCs. We found that CC155 is amongst the most nisin tolerant CCs whereas CC199, CC14, CC403 were amongst CCs with increased nisin sensitivity. Stress response differences among the *L. monocytogenes* genotypes might be linked to differences in expression of several cell membrane-associated genes, including those for penicillin binding proteins (PBPs) (*lmo0441*, *lmo0540*, *lmo1892*, and *lmo2229*) and general stress response gene *sigB*, which were reported to be highly expressed in Lineage II than Lineage I (Severino et al., 2007). Such genes have also been found to be important in nisin responses of *L. monocytogenes* (Gravesen et al., 2004; Begley et al., 2006). Besides that, serotype 1/2a and 1/2c (Lineage II) strains also differ in gene content from serotype 1/2b and 4b (Lineage I) strains (Doumith et al., 2004). The alternative sigma factor *sigC*, that is part of Lineage II specific operon, *lmo0422*-*lmo0423*, was also previously implicated in nisin response (Zhang et al., 2005).

Genome sequence analysis uncovered strain-specific mutations in two genes associated with increased nisin resistance. One mutation involves a non-conserved V240F amino acid substitution in the PBPB3 (Lmo0441) protein. This protein belongs to the class B PBPs that interact with other proteins with different roles including assembly of the peptidoglycan (Goffin and Ghuysen, 1998; Bierne and Cossart, 2007; Korsak et al., 2010), a major constituent of *L. monocytogenes* cell wall (Carvalho et al., 2014; Shen et al., 2017). Increased nisin resistance through PBPs has been previously linked to overexpression of PBPs leading to alteration of cell wall composition and sensitivity to nisin (Gravesen et al., 2001, 2004). The V240F substitution occurs within the PBPB3 allosteric domain (Supplementary Figure S4), which is structurally conserved between *L. monocytogenes* PBPB3 and *S. aureus* PBP2a/MecA (Acebron et al., 2015; Fischer et al., 2020). In *L. monocytogenes*, mutations in this domain were recently linked to increased resistance to the cell envelope targeting antibiotic, cephalosporin (Fischer et al., 2020). Similarly, mutations in *S. aureus* within the same domain have been linked to increased resistance to the cell envelope targeting antibiotics namely ceftaroline and ceftobiprole (Kelley W. L. et al., 2015; Schaumburg et al., 2016; Dafne et al., 2019). It is hypothesized that the PBPB3 allosteric domain is important for cell-wall biogenesis through allosteric regulation, thus mutations within the domain are suggested to interfere with signal transduction between the allosteric domain and the active site (Transpeptidase domain), which potentiate increased resistance to antimicrobial compounds that interfere with peptidoglycan synthesis (Otero et al., 2013; Fishovitz et al., 2014; Acebron et al., 2015). Although future experiments would be needed to prove this, we therefore also hypothesize that the PBPB3_V240F_ mutation might act through similar functional mechanisms to increase nisin resistance as observed in *L. monocytogenes* LK60/1 strain harboring this mutation. We also found that besides increased nisin resistance, the PBPB3 V240F mutant strain also showed increased resistance against the detergent Triton X-100 but its lysozyme resistance was not altered. Like the nisin induced mechanisms, cell death can also occur under detergent stress due to disruption of the structure and increased permeabilization of the cell membrane (Borner et al., 1994; Dayeh et al., 2004). As such mechanisms leading to increased nisin tolerance may have also indirectly increased tolerance to detergent associated stress. Some PBPs have also been previously shown to contribute to virulence in *L. monocytogenes* after disruption of encoding genes through insertion mutagenesis (Guinane et al., 2006a,b). While insertion mutagenesis reduced virulence, our present results showed that the strain harboring the PBPB3_V240F_ mutation had increased virulence in the zebra fish embryo-based infection model. Notably the PBPB3_V240F_ occurred in a CC121 strain, and the CC121 clone is generally regarded as hypovirulent (Maury et al., 2019) suggesting that such a mutation may not only select for nisin tolerant strains in food but also strains with increased virulence.

Another natural mutation we found associated with increased tolerance to nisin among the strains was an RsbU G77S amino acid substitution. On the other hand, there were three strains carrying single base *rsbU* deletion that cause open reading frame shifts and truncation of RsbU *proteins*, which all showed increased nisin sensitivity. Furthermore, there was a 3167 bp deletion affecting *rsbR*, *rsbS*, *rsbT*, *rsbU*, and *rsbV* genes within *sigB* operon detected in another strain displaying increased nisin sensitivity. The role of *rsbU* and *sigB*, in *L. monocytogenes* nisin response has been previously demonstrated (Begley et al., 2006; Palmer et al., 2009; Shin et al., 2010). *sigB* is activated by a signal transduction cascade involving RsbR, RsbS, RsbT, RsbU, RsbV, and RsbW proteins (Guldimann et al., 2016). In particular, *L. monocytogenes* response to nisin stress through *sigB*, relies on the phosphorylation of RsbU protein, which is a serine phosphatase and its absence was linked to increased nisin sensitivity (Shin et al., 2010). It is plausible but not yet proven that the G77S substitution might cause increased RsbU phosphorylation leading to increased activation of SigB and its regulon thereby indirectly promoting increased resistance to general stress including nisin. Increased SigB regulon activation in the RsbU G77S mutant was supported by the higher mRNA levels for the SigB-dependent gene *gadD3* detected in this mutant compared to a control strain from the same MLST CC without this RsbU amino acid substitution. *gadD3* is positively regulated by SigB (Raengpradub et al., 2008) and constitutes part of the GAD system, which is also involved in nisin response (Begley et al., 2010; Szendy et al., 2019). In contrast, the nonsense mutation and in-frame deletions in *rsbU* gene could have resulted in the coding of a functionally impaired RsbU protein and the deletion of the five genes in the *sigB* operon may also have impaired the signal transduction, which is important for activation of SigB and its regulon. Consistent with impaired SigB activity we similarly detected low *gadD3* mRNA expression levels in all these *sigB* operon mutants compared to a corresponding control strain from the same CC but without such mutations. Although not investigated here, SigB also regulates *nag* operon (*lmo956*–*lmo958*) required for the synthesis of cell wall peptidoglycan and teichoic acid, and consequently, response to cell wall targeting hydrolases and antimicrobials (Popowska et al., 2012; Guariglia-Oropeza et al., 2014). The activation of these genes might also be indirectly increased in the RsbU G77S mutant providing a possible explanation for the increased lysozyme tolerance observed in the nisin resistant strain harboring this mutation. Lysozyme acts on bacterial cell wall by hydrolyzing bacterial peptidoglycans (Wohlkönig et al., 2010; Primo et al., 2018). Interestingly, in this case there was a direct relationship between the response to nisin and Triton X 100 among the strains with mutations in the *sigB* operon, which may indicate the mutations, among other effects, might have affected cell membrane composition and/or permeability. While the role of *sigB* operon in *L. monocytogenes* virulence has been established through studies mainly targeting the *sigB* gene (Kazmierczak et al., 2003), our present results indicate that the signal transduction induced by *rsbU* during stress response by the *sigB* operon might also have an impact on virulence. We show here a correlation between nisin tolerance and zebra fish virulence phenotypes among the *rsbU* variant strains showing increased nisin resistance and sensitivity. Similarly, *rsbT* and *rsbV*, which occur upstream and downstream of *rsbU*, respectively, were postulated to contribute to the *sigB* dependent regulation of virulence in *L. monocytogenes* (Chaturongakul and Boor, 2004; Zhang et al., 2013).

As might be expected the natural mutations within *dlt* operon including deletion in *dltA* and its promoter as well as missense *dltB* and *dltD* mutations were all associated with increased nisin sensitivity. These three genes are part of the *dltABCD* operon, which among other functions is also important for nisin resistance in *L. monocytogenes* and other Gram-positive bacteria (Abachin et al., 2002; Weidenmaier et al., 2005; Fisher et al., 2006; Kramer et al., 2006; McBride and Sonenshein, 2011). Respectively, the *dltABCD* genes code for DltA, a D-alanyl carrier protein ligase, DltB, a transmembrane protein that effluxes D-alanine to incorporation sites, DltC, a D-alanine carrier protein and DltD, a multifunctional membrane protein, all of which are required for the decoration of Gram positive bacteria cell wall lipoteichoic acid (LTA) with D-alanyl (Perego et al., 1995; Neuhaus et al., 1996; Debabov et al., 2000). The incorporation of D-alanine into the LTA increases the positive charges in the otherwise negatively charged cell wall, which in-turn repels the positively charged cationic antimicrobials, including nisin and prevents them from reaching their primary targets, including lipid II in the case for nisin (Nawrocki et al., 2014; Zhou et al., 2014). Therefore, the mutations in the *dlt* operon may have interfered with the teichoic acid D-alanylation pathway thus increasing the affinity of nisin and the cell wall consequently sensitizing the mutants to the antimicrobial compound. The absence of D-alanine in the cell wall was previously demonstrated to increases the nisin susceptibility of *L. monocytogenes* (Abachin et al., 2002). Increased cytochrome c binding an indication of a more negatively charged cell envelope (Ernst et al., 2009), was demonstrated in all the nisin sensitive *dlt* operon mutant strains discovered in our study when compared to corresponding un mutated control strains from the same MLST CC. The increased electronegative charges in a *dltA* null mutant were previously linked to reduced virulence in *L. monocytogenes* due to low adherence to non-phagocytic and phagocytic cells (Abachin et al., 2002). Similarly, we demonstrated here that a natural mutant without the *dltA* promoter and an N-terminal 66 amino acid DltA deletion is less virulent toward zebra fish compared to a control strain with an intact *dltA* gene and *dlt* operon promoter.

Nisin sensitive strains also carried mutations within *vir* operon including missense and protein truncating mutations of *virR* and *virB* genes. VirR is a response regulator of the VirRS two component system, whereas VirB is part of the VirAB ABC transporter system, which is also involved in VirRS dependent nisin sensing (Mandin et al., 2005; Grubaugh et al., 2018). The VirABRS proteins are thus involved in nisin and cell envelope stress response through sensing nisin and regulating the transcription of genes in VirR regulon that include *mprF* and *dltABCD* (Sun et al., 2009; Jiang et al., 2019). MprF, like the DltABCD proteins also increases the positive charges within the cell envelope by incorporating lysine into the membrane’s diphosphatidylglycerol (Thedieck et al., 2006). The regulation of *mprF* and *dltABCD* gene expression through VirR is partly dependent on VirB, hence mutations within these two genes in nisin sensitive strains detected may have interfered with the sensing of nisin by VirB and regulation of multiple VirR-dependent nisin response genes. In support of this hypothesis downregulation of *mprF* and *dltA* expression as well as increased sensitivity to bacitracin was detected in the *virR* and *virB* nisin sensitive mutants from our study. On the other hand, the VirB and VirR form a pathway that regulates virulence genes in addition to the nisin resistance genes. We showed that the VirB_G120D_ mutant strain also caused significantly less zebrafish virulence compared to control strain harboring a normal wild type VirB variant. A possible explanation for the reduced functionality due to the VirB_I1__17__T_ amino acid substitution mutation might be predicted disruption the normal VirB protein structure arising from the covalent bond predicted between Tyr^124^ and Ile^169^ residues in the VirB I128T mutant protein.

## Conclusion

Combining nisin response phenotype evaluation and targeted gene sequence analysis allowed identification of naturally occurring genotypes and mutations among 356 *L. monocytogenes* field strains linked to increased and reduced tolerance to nisin applied at 12.5 ppm, a concentration widely used in food processing. We showed that natural phenotypic nisin response variation exists at genotype and strain-level as well as depending on the isolation sources of the strains. Natural RsbU_G77S_ and PBPB3_V240F_ mutations were discovered among strain showing increased nisin resistance. The RsbU_G77S_ mutation predicted to induce RsbU protein structural changes was associated with increased SigB activity as well as detergent and lysozyme stress resistance. Both RsbU_G77S_ and PBPB3_V240F_ increased nisin resistance strains also showed increased virulence as judged through a zebra fish embryo-based infection model. Nisin sensitive strains were associated with several natural mutations including missense, nonsense and deletions mutations affecting *sigB*, *vir* and *dlt* operon genes. Impaired SigB activity was confirmed in all *sigB* operon mutation harboring strains as they showed reduced expression of SigB-dependent *gadD3* mRNA as well as reduced resistance against detergent and lysozyme stress. Nisin sensitive strains with *dlt* operon mutations also showed reduced lysozyme resistance and altered cell surface charge consistent with altered Dlt system functions. Strains harboring *vir* operon mutations showing increased nisin sensitivity were confirmed to have impaired VirR-dependent functions since they showed increased bacitracin sensitivity and reduced expression of the VirR-dependent genes *dltA* and *mprF*. We showed that strains with either deletion in the *dltA* gene and its promoter, VirB_G120D_ amino acid substitution or premature stop codon in RsbU were all reduced in virulence. We showed through identification and further phenotypic analysis of these natural occurring mutation of known nisin response genes that a combination phenome assessment based on the AUC growth parameter in combination with genome sequence analysis is an effective approach to identify nisin response genes. Employing this technique in future we aim to identify novel nisin response genes and alleles in nisin tolerant and nisin sensitive strains in which mutations in known genes were not identified. While our study has provided genomic evidence for associating naturally occurring nisin response gene mutations described here with increased and reduced nisin sensitivity phenotypes among *L. monocytogenes* field strains covering different genetic background further experimental work in future will be necessary to provide functional confirmation and validation of the link between such genomic changes and the altered nisin tolerance phenotypes uncovered in our study.

## Data Availability Statement

The datasets presented in this study can be found in online repositories. The names of the repository/repositories and accession number(s) can be found in the article/ Supplementary Material.

## Ethics Statement

Ethical review and approval was not required for the animal study because the maximum age reached by the zebrafish embryos used during experimentation was 5 days post fertilization (dpf) for which no license is required from the Cantonal Veterinary Office in Switzerland, since such embryos will not have yet reached the free-feeding stage.

## Author Contributions

TT and JW designed the study. TT supervised the study. JW and AE performed the experiments. JW, PN, and MS performed the biostatistical and data analyses. MA-A and AP performed the whole-genome sequencing and compiled the genomic data. JW and TT wrote and revised the manuscript. All the authors contributed to the article and approved the submitted version.

## Conflict of Interest

The authors declare that the research was conducted in the absence of any commercial or financial relationships that could be construed as a potential conflict of interest.

## Footnotes

1 https://www.ncbi.nlm.nih.gov/

2 http://rast.nmpdr.org/

3 https://blast.ncbi.nlm.nih.gov/Blast.cgi

4 https://www.ncbi.nlm.nih.gov/Structure/cdd/wrpsb.cgi

5 https://bigsdb.pasteur.fr/listeria/

6 http://tree.bio.ed.ac.uk/software/figtree/

7 http://www.sbg.bio.ic.ac.uk/~missense3d/
