# Supplementary material for: The Analysis of Field Strains Isolated From Food, Animal and Clinical Sources Uncovers Natural Mutations in Listeria monocytogenes Nisin Resistance Genes
Source: Front Microbiol. 2020 Oct 6;11:549531. doi: 10.3389/fmicb.2020.549531 (PMC7574537; doi:10.3389/fmicb.2020.549531)
Supplement: Supplementary file 5 [file Data_Sheet_1.PDF]

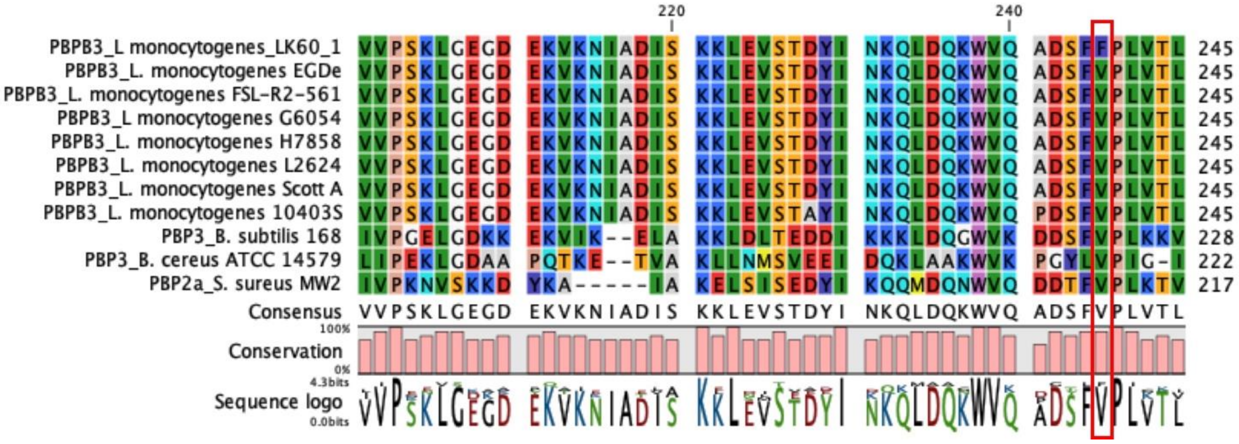

Supplementary FIG S1. PBPB3 (Lmo0441) protein sequence alignments of nisin tolerant *L. monocytogenes* strain LK60/1 with homologous wild type proteins of representative *L. monocytogenes*, *Bacillus cereus*, *Bacillus subtilis* and *Staphylococcus aureus* strains. Compared to the proteins of the reference strains, a $PBPB3_{V240F}$ amino substitution was detected in the nisin resistant strain affecting a conserved $Valine^{240}$ residue.

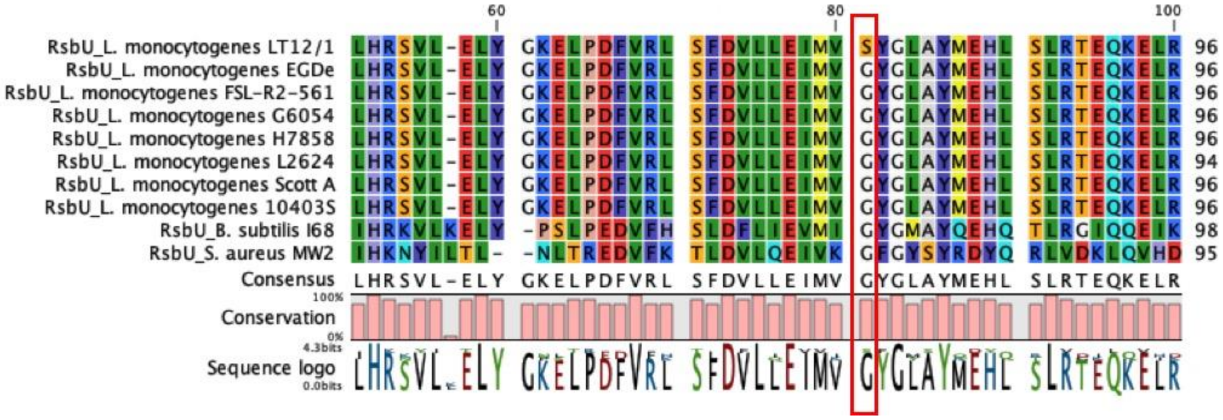

Supplementary FIG S2. RsbU protein sequence alignments of nisin tolerant *L. monocytogenes* strain LT12/1 with homologous wild type RsbU proteins of representative *L. monocytogenes*, *Bacillus subtilis* and *Staphylococcus aureus* strains. Compared to the proteins of the reference strains, a RsbU G77S amino substitution in a conserved $Glycine^{77}$ residue was detected in the nisin resistant strain.

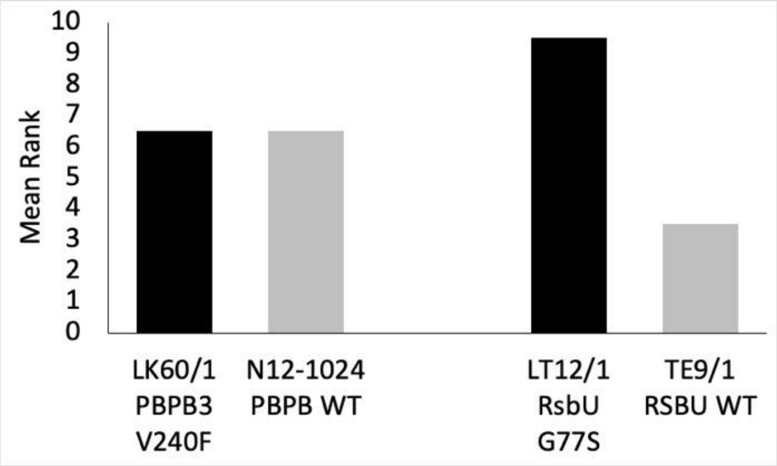

Supplementary FIG S3. Evaluation of lysozyme response in high nisin resistant *L. monocytogenes* strains with natural $PBPB3_{V240F}$ and $RsbU_{G77S}$ amino substitutions. The $PBPB3_{V240F}$ mutant strain (LK60/1) had similar response to its control strain (N12-1024) while the $RsbU_{G77S}$ mutant (LT12/1) had a significantly higher resistance to lysozyme ($P<0.05$) compared to its control strain (TE9/1). Significant differences were identified using the non-parametric Wilcoxon signed-rank test ($P=0.05$) between the mutants and the respective control strains.

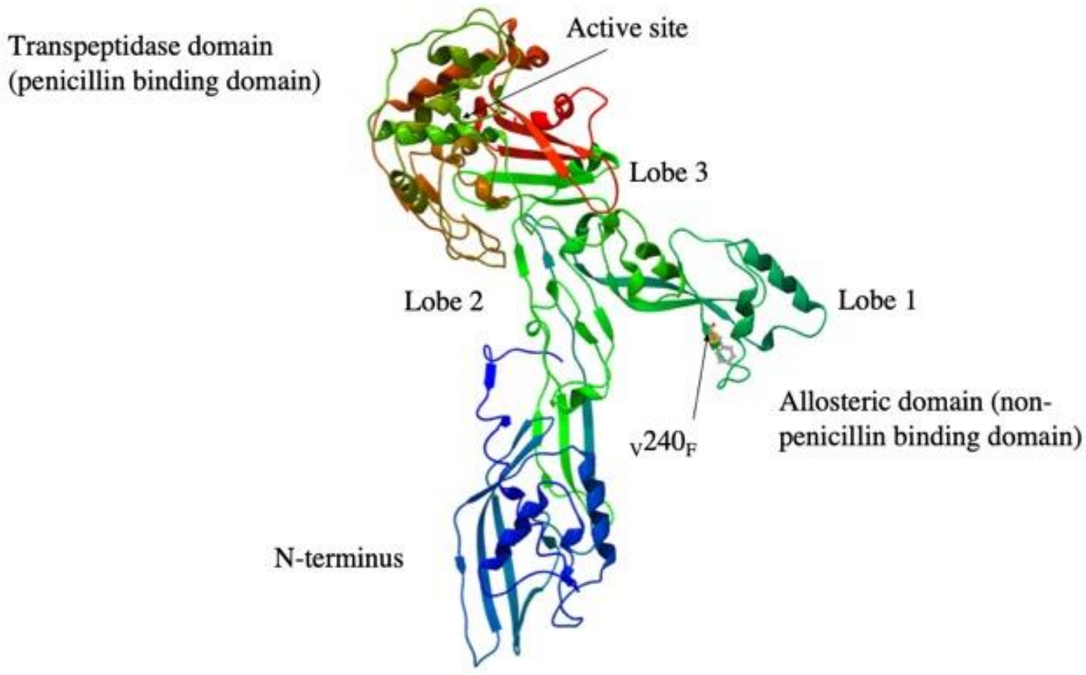

Supplementary FIG S4. Structural predictions of *L. monocytogenes* PBPB3 with the V240F mutation. The mutation occurs in the Allosteric domain (previously referred to as the non-penicillin binding domain). The Figure was predicted in Phyre2 webservers and visualized in CLC Genomics Workbench

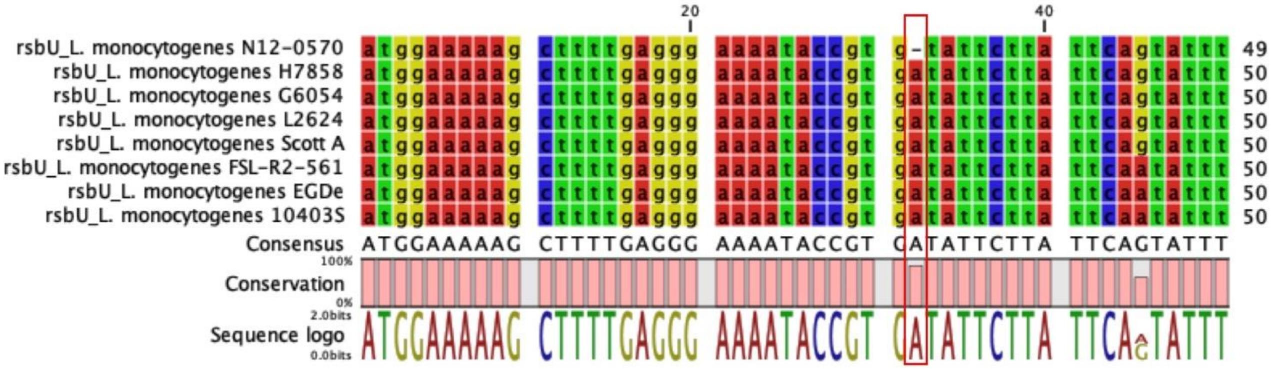

Supplementary FIG S5. Genome sequence alignment of *rsbU* (*lmo0892*) mutant genes in nisin sensitive *L. monocytogenes* N12-0570 strains with homologous wild type genes from *L. monocytogenes* representative strains. The strain had an indel of an adenine at 32 bp.

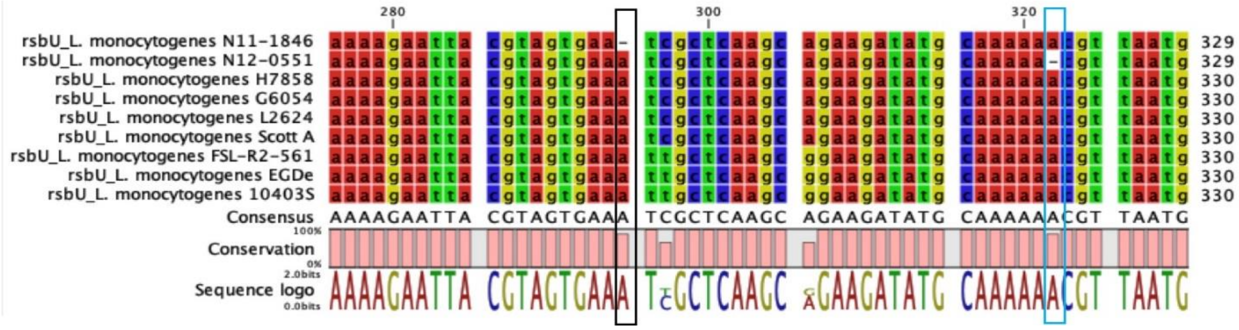

Supplementary FIG S6. Genome sequence alignment of *rsbU* (*lmo0892*) mutant genes in nisin sensitive *L. monocytogenes* N11-1846 and N12-0551 strains with homologous wild type genes from *L. monocytogenes* representative strains. Adenines at 295 (black) and 322 (blue) bp in *L. monocytogenes* N11-1846 and N12-0551 strains, respectively, have been naturally deleted.

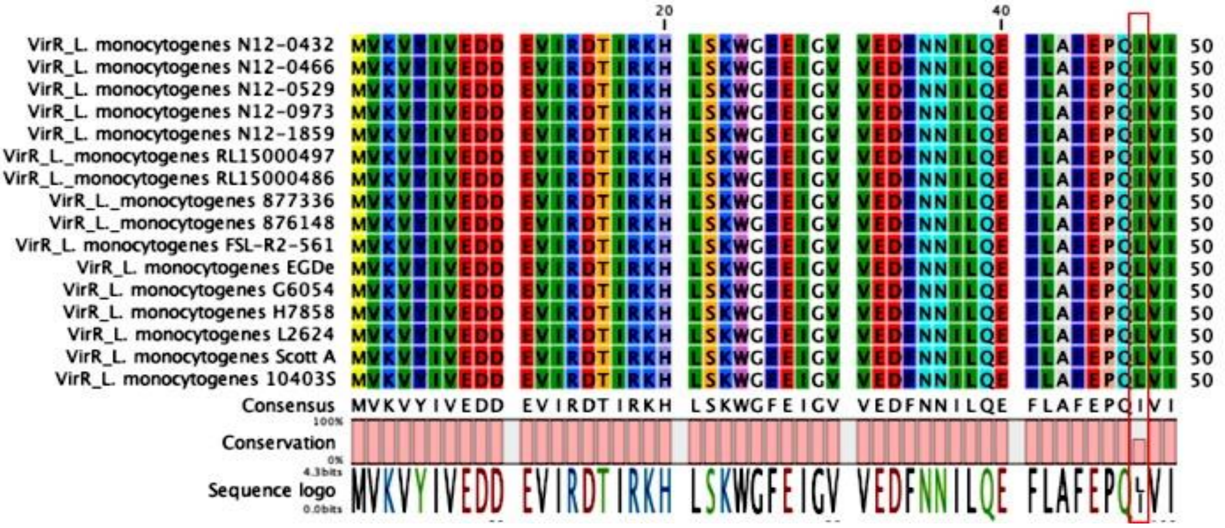

Supplementary FIG S7. VirR protein sequence alignments of nisin sensitive *Listeria monocytogenes* strains N12-0432, N12-0466, N12-0529, N12-0973 and N12-1859 with homologous VirR wild types of representative *L. monocytogenes* reference strains. VirR$_{L48I}$ amino acid substitution mutation was identified in all the five nisin sensitive strains and four other strains *L. monocytogenes* strains RL15000497, RL15000486, 877336 and 876148 from the NCBI database compared to the conserved VirR Leucine^{48} in representative *L. monocytogenes* reference strains.

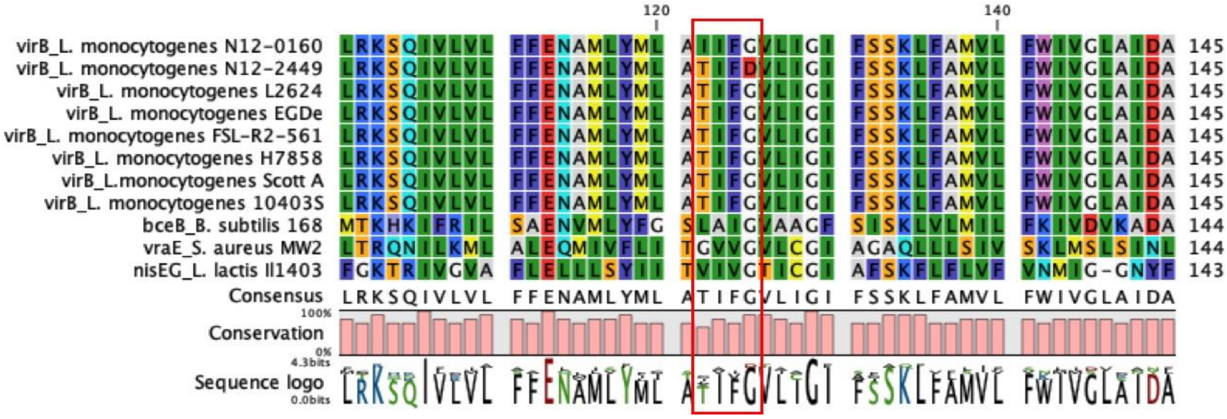

Supplementary FIG S8. VirB protein sequence alignments of nisin sensitive *Listeria monocytogenes* strains N12-0160 and N12-2449 with homologous wild type proteins of representative *L. monocytogenes*, *Bacillus subtilis*, *Staphylococcus aureus* and *L. lactis* reference strains. VirB$_{T117I}$ and VirB$_{G120D}$ amino acid substitution mutations were identified in strains N12-0160 and N12-2449, respectively compared to homologous wild type proteins of representative reference strains.

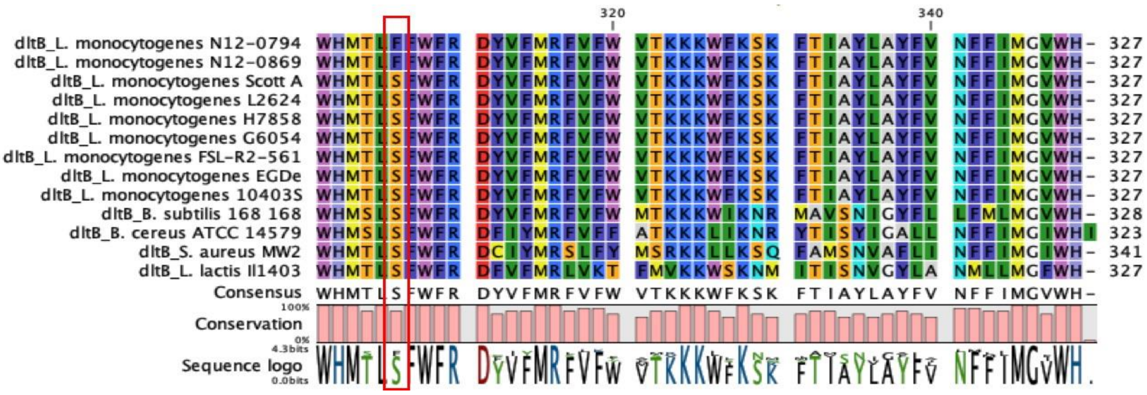

Supplementary FIG S9. DltB protein sequence alignments of nisin sensitive *Listeria monocytogenes* strains N12-0794 and N12-0869 and homologous wild type DltB proteins from *L. monocytogenes*, *Bacilus cereus*, *B. subtilis*, *Staphylococcus aureus* and *Lactococcus lactis* representative strains. A $DltB_{S284F}$ amino acid substitution mutation was detected the nisin sensitive *L. monocytogenes* strains N12-0794 and N12-0869 compared to homologous wild type DltB proteins from the reference strains.

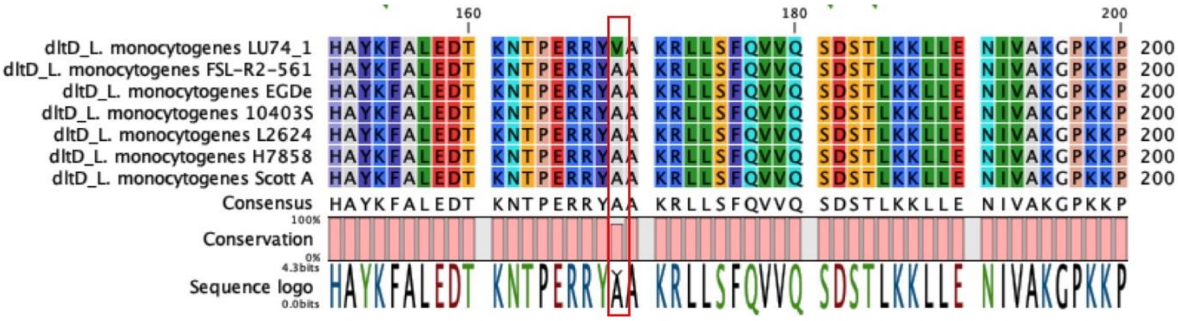

Supplementary FIG S10. DltD protein sequence alignments of nisin sensitive *L. monocytogenes* strain LU74/1 with homologous DltD protein wild types of representative *L. monocytogenes* strains. Compared to the reference strains, a $DltD_{A69V}$ amino substitution was detected in the nisin sensitive strain in a conserved Alanine of the reference strains.

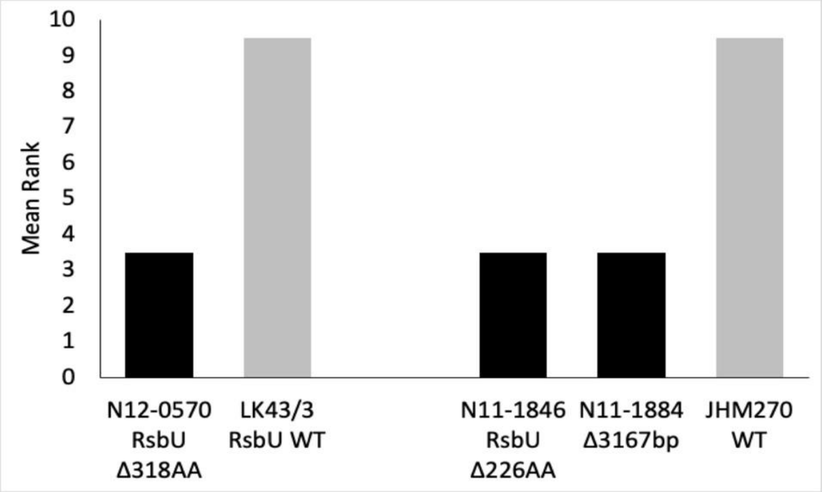

Supplementary FIG S11. Evaluation of lysozyme response in high nisin sensitive *L. monocytogenes* strains with natural *sigB* operon mutations. Nisin sensitive sigB operon mutant strains N12-0570 ($RsbU_{\Delta318AA}$), N11-1846 ($RsbU_{\Delta226AA}$) and N11-1884 (Δ3167 bp) were more sensitive to lysozyme ($P<0.05$) compared to their corresponding clonal control strains LK43/3 and JHM270. Significant differences were identified using the non-parametric Wilcoxon signed-rank test ($P=0.05$) between the mutants and the respective control strains.
